# Simultaneous detection of lymphocytes and tumor cells *in vivo* in response to STING-TLR9 immunotherapy with Raman active multiplexed gold nanostars

**DOI:** 10.1039/d5nh00687b

**Published:** 2026-01-07

**Authors:** Siddhant Kothadiya, Gabriel P. Cutshaw, Ansuja P. Mathew, Casey Zielinski, Rizia Bardhan

**Affiliations:** a Department of Chemical and Biological Engineering, Iowa State University Ames IA 50011 USA rbardhan@iastate.edu; b Nanovaccine Institute, Iowa State University Ames IA 50012 USA

## Abstract

Immunotherapies show heterogeneous response in patients and identifying those likely to benefit from these therapies remains challenging. This is in part because histopathology, the current clinical standard, cannot accurately predict response. Dynamic changes occur in both tumor cells and immune cells *in vivo* during and after treatment which are not captured by histopathology or by single biomarker imaging. To address this urgent need, this study leverages multiplexed profiling of both CD8^+^ T cells and VEGFR2^+^ expressing tumor cells in 4T1 murine breast cancer tumors with surface-enhanced Raman spectroscopy (SERS) using multiplexed gold nanostars (MGNs). MGNs are conjugated with antibodies targeting each cell type and Raman labels to enable multiplexing. Real time SERS *in vivo* imaging enables detection of dynamic longitudinal changes in CD8 and VEGFR2 in response to STING + TLR9 (stimulator of interferon genes + toll like receptor 9) immunotherapies, a treatment that increases tumor immunogenicity through a type I interferon response. MGNs also distinguished nonresponders of immunotherapies where 4T1 tumors were treated with antiOX40 antibodies. *In vivo* endpoints were validated *ex vivo* with flow cytometry analysis of immune cell population, cytokine analysis, STING activation, and immunofluorescence (IF) imaging of key markers (CD8, VEGFR, CD31, Ki67, and STING). Further, high resolution SERS maps provided a spatial context of CD8 and VEGFR2 distribution that showed the molecular makeup of tumors in responder and nonresponder mice. Biomarker distribution in *ex vivo* SERS aligned with *in vivo* findings and showed moderate to strong correlations *via* a Pearson's correlation to quantification of IF markers in tumors.

## Abstract 2

Multiplexed gold nanostars enable *in vivo* and *ex vivo* SERS imaging of CD8^+^ T cells and VEGFR2^+^ tumor cells capturing dynamic T cell recruitment and vascular changes in the tumor to enable treatment response to immunotherapy.

## Graphical abstract

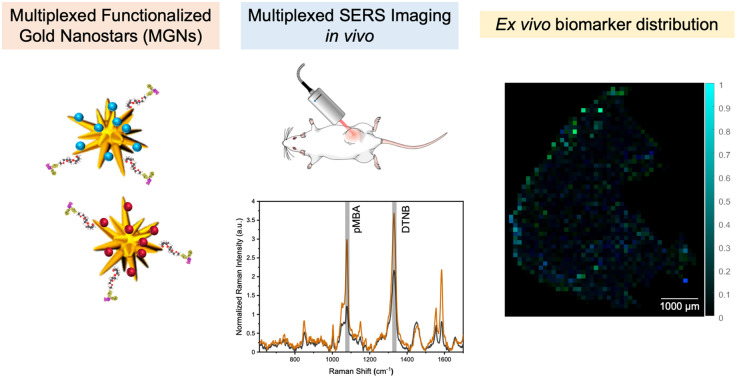

**New concepts**

Here, we report the development of multiplexed gold nanostars (MGNs) engineered for real time *in vivo* detection of key immune and angiogenic markers in the tumor microenvironment (TME). The key conceptual leap in our approach is the simultaneous, real-time, and spatially resolved multiplexing capability *in vivo*. Anisotropic MGNs enable highly sensitive SERS multiplexed monitoring of VEGFR2 downregulation and CD8^+^ T cell infiltration in immunologically cold 4T1 tumors in response to STING + TLR9 immunotherapy. Further, MGNs also distinguish responders from nonresponders of therapy. This approach integrates *in vivo* imaging with *ex vivo* SERS mapping of tumor sections to achieve cellular-level spatial resolution of distinct cell types in the TME. By tracking vascular changes and immune cell trafficking in the same tumor, our approach surpasses clinical imaging approaches that does not support multiplexing (MRI, PET), as well as other nanoparticle-based imaging where photobleaching and autofluorescence dampens signal *in vivo.*

## Introduction

Dynamic, real-time molecular imaging *in vivo* of key oncogenic markers in the tumor microenvironment (TME) has transformed our ability to understand the origins and vulnerabilities of cancer, and to evaluate response to innovative therapies early in the treatment regimen.^1^ Conventional imaging and histopathology approaches often fail to predict response or resistance in part due to their inability to accurately profile heterogeneous tumors,^2^ and capture dynamic changes that occur in different cell types during treatment. Therefore, molecular imaging techniques that surpass these limitations are indispensable to enable multiplexed detection *in vivo* and facilitate noninvasive longitudinal tracking of multiple cells in the tumor milieu.^3^ This emerging need has propelled the design of nanoscale molecular probes that are biocompatible, stable *in vivo*, have long systemic circulation, can selectively accumulate in the TME, and enable highly sensitive and specific imaging by actively targeting desired markers in response to treatment.^4,5^ In the past decade, molecular imaging with both small-molecule and nanoscale probes has made a tremendous impact on immunotherapy response^6^ enabling patient selection of those who are likely to respond to immunotherapies.^7^

Among immunotherapy targets, toll-like receptors (TLRs) that serve as a bridge between innate and adaptive immune responses have shown favorable outcomes in patients.^8,9^ TLRs are expressed either on the cell surface or within endosomes and act as cellular sentinels recognizing danger signals including pathogen-associated molecular patterns and damage-associated molecular patterns.^10^ The binding of these danger signals to TLRs triggers the activation of dendritic cells (DCs) that stimulates pro-inflammatory responses and cytokine secretion; this is followed by differentiation of naive T lymphocytes into effector T cells. Several synthetic ligands for TLR9 including nucleic acid-derived immunoadjuvants, such as CpG oligodeoxynucleotides (CpG ODNs), have served as potent immunostimulants that activate NF-κB and other downstream pathways, initiate the production of type 1 interferons (IFN-1), and elicit cytotoxic CD8^+^ T cell responses in the TME.^11–13^ Recent findings also show TLR9 agonists when combined with stimulator of interferon genes (STING) agonists potentiate high therapeutic benefit specifically in immunologically cold tumors that are nonresponsive to conventional treatments and most immunotherapies.^14,15^ STING, an immunostimulatory molecule, is the key cytosolic sensor of cyclic dinucleotides and plays an essential role in cancer immune surveillance.^16,17^ Activation of STING stimulates the host innate immunity through IFN-1 production and mediates a signaling cascade *via* the TBK1-NFκB pathways increasing tumor immunogenicity. This process enables DC maturation and activation, as well as cross-presentation of the tumor antigens, thus recruiting cytotoxic T cells to the TME.^18,19^ However, STING agonists alone induce pro-tumorigenic type-2 rather than anti-tumor type-1 immune responses, which limits their therapeutic potential^20^ and motivates the synergistic combination with TLR9 agonists to serve as a potent type-1 adjuvant, amplify innate and adaptive IFN-γ production, and significantly suppress tumor growth.

In this work we demonstrate early response to STING + TLR9 combination immunotherapy in an immunologically cold 4T1 breast cancer (BC) murine tumor model with multiplexed dynamic molecular imaging with surface-enhanced Raman spectroscopy (SERS). Raman spectroscopy (RS) is an optical technique based on the inelastic scattering of light, where shifts in the frequency of incident light correspond to the vibrational modes of chemical bonds in molecules.^21^ The RS signal *in vivo* can be substantially amplified to enable longitudinal tracking of subtle changes in the tumor milieu by conjugating Raman labels to morphology-controlled gold nanostars (GNs). The anisotropic shape of GNs generates high electromagnetic near-fields along the protrusions giving rise to electromagnetic enhancements.^22^ The covalently-bound Raman labels also enable electron transfer with GNs that results in chemical enhancement further amplifying the SERS effect. The two enhancement mechanisms in conjunction allow GNs to track multiple cell types simultaneously *in vivo* in the TME *via* SERS. GNs are within the desired size range for efficient tumor penetration, are biocompatible and break down *in vivo* that is amenable to rapid clearance,^23^ and has shown efficacy as a therapeutic^24,25^ and diagnostic agent.^26–28^ Here multiplexed GNs (MGNs) were designed by covalently attaching monoclonal antibodies (mAbs) targeting CD8^+^ T cells and vascular endothelial growth factor receptor 2 (VEGFR2^+^) tumor cells, and two different Raman labels associated with each cell type. VEGF is a key angiogenic marker in the TME as rapidly proliferating tumor cells require a well-vascularized system to meet their increased nutritional and oxygen demand. VEGF promotes endothelial cell survival, proliferation, and migration and thus serves as a primary regulator of tumor neovascularization.^29,30^ VEGF binds to its receptor tyrosine kinases, VEGFR1 and VEGFR2, and the latter is found to be overexpressed in human BC and in 4T1 murine tumors that underscores longitudinal tracking of VEGFR2 in the immune TME.^31^ Further, activation of STING/TLR9 pathway with agonists normalizes the tumor vasculature that synergizes with VEGFR2 blockade,^32^ and also downregulates VEGFR expression in mouse models.^32,33^ Here we show that dynamic changes occur in 4T1 tumors, involving both CD8^+^ T cell recruitment in the TME and reduction in VEGFR2^+^ expression in response to STING + TLR9 immunotherapy that is captured by longitudinal SERS *in vivo* with MGNs. We also show that MGNs can identify resistance to treatment by tracking these markers in 4T1 tumors when treated with antiOX40 immunotherapy. *In vivo* SERS endpoints were validated with immune profiling using flow cytometry analysis (FACS), quantitative immunofluorescence (IF) of tissues, measuring pro-inflammatory cytokines with Luminex, and IFN-1 response with enzyme-linked immunosorbent assay (ELISA). Our findings show that multiplexed real-time tracking *via* SERS with MGNs has the potential to ultimately advance the drug development pipeline by identifying patients who are most likely to benefit from specific immunoagents and reduce the costs associated with ineffective therapies.

## Results and discussions

**Fig. 1:**
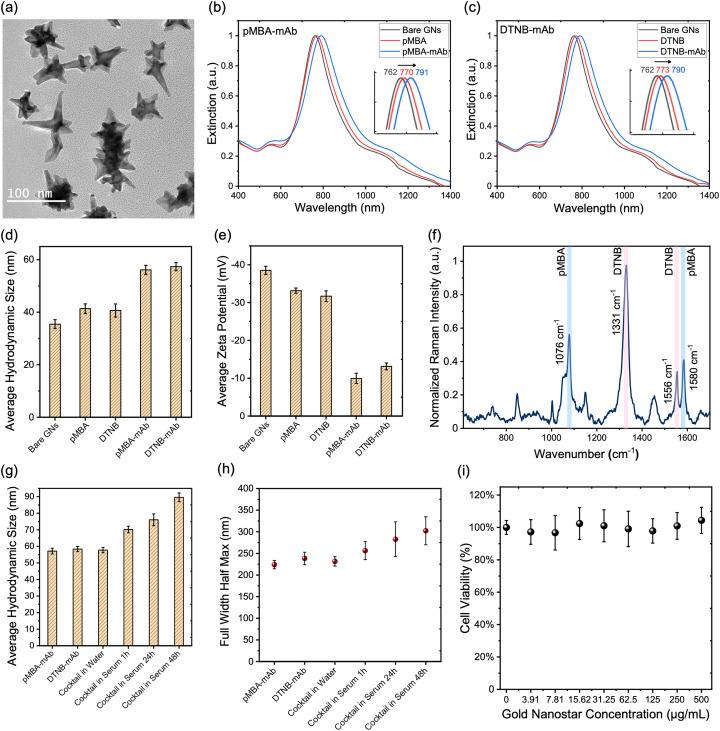
Characterization of multiplexed gold nanostars (MGNs). (a) Transmission electron micrograph of GNs showing their anisotropic morphology. (b) and (c) Extinction spectra of bare GNs and GNs functionalized with (b) pMBA Raman label and antibody (c) DTNB Raman label and antibody. (d) Hydrodynamic size of GNs and after functionalizing with Raman labels and antibody obtained with dynamic light scattering measurements (*n* = 8) and (e) corresponding zeta potential measurements showing surface charge (*n* = 8). (f) SERS spectra of MGNs (GNs/anti-VEGFR2/pMBA + GNs/anti-CD8/DTNB at a 1 : 1 ratio) targeting both VEGFR and CD8 *via* DTNB (1331 cm^−1^) and pMBA (1076 cm^−1^) reporters, respectively; the primary and secondary peaks of the reporters are highlighted. (g) Hydrodynamic size of MGNs before and after incubation with 60% fetal bovine serum (FBS), and (h) corresponding full width at half maximum (FWHM) of the extinction of the same samples to examine serum stability (*n* = 5). (i) Concentration dependent CCK-8 cell viability assay of MGNs in 4T1 cells after 24h incubation showing biocompatibility.

GNs were synthesized through a one-step seedless method, using a 2-[4-(2-hydroxyethyl)piperazin-1-yl]ethanesulfonic acid (HEPES) buffer at pH 7.4 as both a capping and reduction agent to promote GN growth.^22^ GNs were then covalently conjugated with Raman labels that have thiol bonds including *para*-mercaptobenzoic acid (pMBA) and 5,5′-dithiobis(2-nitrobenzoic acid) (DTNB) to enable strong SERS signal *in vivo*. Targeted detection of CD8^+^ T cells and VEGFR2^+^ tumor cells *in vivo* was achieved through functionalization of Raman label conjugated GNs with monoclonal antibodies (mABs) *via* a bifunctional linker OPSS-PEG-SVA (*ortho*-pyridyl disulfide polyethylene glycol succinimidyl valerate, 2000 kDa). This resulted in the final multiplexed gold nanostars (MGNs) which is a 1 : 1 mixture of GNs/anti-VEGFR2/pMBA + GNs/anti-CD8/DTNB that would be used *in vivo*. The fully conjugated MGNs were characterized with transmission electron microscopy (TEM) to confirm GNs morphology (Fig. 1a) and overall extinction was measured that showed ∼30 nm redshift over the functionalization process (Fig. 1b and c). A ∼10 nm shift was observed following Raman label functionalization, with an additional ∼20 nm shift after antibody conjugation. The final extinction of the MGNs remained resonant with the 785 nm NIR excitation laser used for SERS imaging *in vivo*. MGNs overall hydrodynamic size was confirmed with Dynamic light scattering (DLS) which changed from initial ∼40 nm to ∼65 nm following full functionalization (Fig. 1d). MGNs surface charge was reduced from −40 mV to near-neutral at −10 mV after functionalization, due to the presence of PEG in the OPSS-PEG-SVA linkers, improving MGNs biocompatibility for *in vivo* delivery (Fig. 1e). SERS spectra of MGNs (GNs/anti-VEGFR2/pMBA + GNs/anti-CD8/DTNB at a 1 : 1 ratio) showed that both Raman labels are easily visualized (Fig. 1f) with minimal spectral overlap which includes the primary peaks (pMBA 1076 cm^−1^ and DTNB 1331 cm^−1^) and secondary peaks (pMBA 1580 cm^−1^ and DTNB 1556 cm^−1^) of both labels. The serum stability of the functionalized MGNs was assessed in 60% fetal bovine serum (FBS) by mixing GNs/anti-VEGFR2/pMBA + GNs/anti-CD8/DTNB at 1 : 1 ratio with FBS and rotated at 100 rpm at 37 °C to simulate *in vivo* conditions. After incubation, samples were collected at each timepoint (1 h, 24 h, 48 h), purified and resuspended in water, and size of MGNs was measured with DLS (Fig. 1g) and the full width at half maximum (FWHM) of the extinction spectra was measured (Fig. 1h). Large variations in overall size and FWHM typically indicate nanoparticle instability and aggregation. Our results show minimal increase in size and FWHM after incubation in serum for 24 h indicating good stability of MGNs without significant agglomeration over the tested periods. Note that we examined serum stability of MGNs that were functionalized with a final layer of mono-functionalized PEG-thiol, but we did not see a benefit in overall stability, and it dampened the SERS signal due to steric hindrance of PEG layers over the small Raman labels (SI Fig. S1). Therefore, for *in vitro* and *in vivo* experiments a final PEG conjugation was not pursued. In addition to serum stability, we evaluated the shelf-life of the MGNs cocktail under standard storage conditions. Concentrated MGNs were aliquoted in water and stored at 4 °C for up to 10 days, corresponding to typical short-term storage conditions relevant for batch production and clinical use. At each timepoint, aliquots were centrifuged to remove any loosely associated aggregates prior to analysis. Stability was assessed by measuring the hydrodynamic diameter using DLS and the FWHM of the extinction spectra. Minimal variation in particle size and FWHM was observed over the storage period, indicating that MGNs maintain colloidal and optical stability during short-term refrigerated storage (SI Fig. S2). The biocompatibility of MGNs was assessed *in vitro* in 4T1 cells using the CCK-8 cell viability assay (Fig. 1i). Cell viability was determined by subtracting blank values and comparing the results to cells cultured in untreated medium. Minimal changes in the viability were observed across the range of concentrations tested that confirmed that MGNs have minimal cytotoxicity in cells. Before conducting experiments *in vivo*, we also wanted to examine the efficacy of our combination immunotherapy in 4T1 cells combining STING agonist (ADU S100) + TLR9 agonist (ODN 2395) treatment. A CCK-8 cell viability assay of this treatment combination indicated strong therapeutic response and resulting cancer cell death in a dose-dependent manner (SI Fig. S3).

**Fig. 2:**
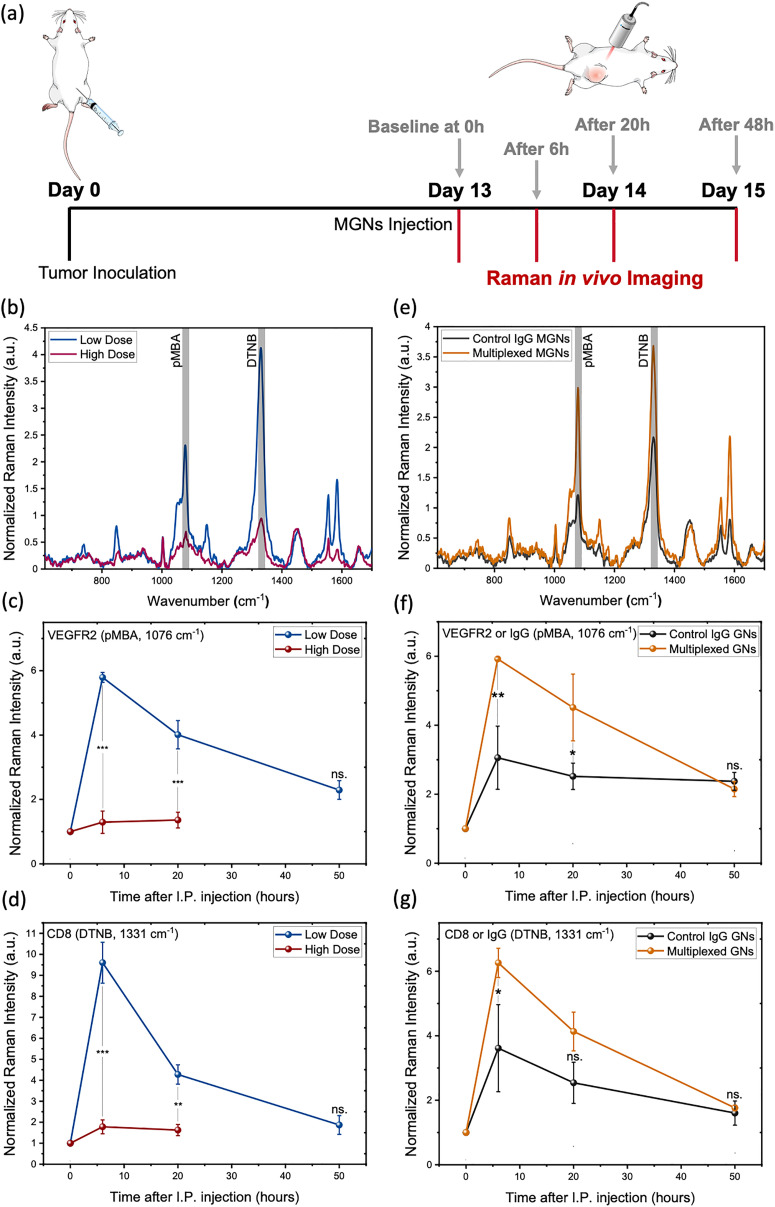
SERS imaging *in vivo* with multiplexed gold nanostars (MGNs) – dosing and specificity study. (a) Schema of SERS imaging timeline *in vivo*. (b)–(d) Dose dependence study where mice received either high dose (1.2 mg) (*n* = 5) or low dose (0.6 mg) (*n* = 5) MGNs (GNs/anti-VEGFR2/pMBA + GNs/anti-CD8/DTNB at a 1 : 1 ratio) *in vivo* in 4T1 tumor model. (b) *In vivo* SERS spectra at 6 h post injection of MGNs at low (blue) and high (maroon) dose. (c) and (d) Longitudinal signal throughout tumor between the two doses tracked for the (c) pMBA signal at 1076 cm^−1^ (conjugated with anti-VEGFR2 mAb) (d) DTNB signal at 1331 cm^−1^ (conjugated with anti-CD8 mAb). Please note that due to significant aggregation at the high dose of MGNs, we observed significant signal dampening and therefore good signal-to-noise could not be achieved at the 50 h timepoint. (e)–(g) Comparing the specific targeting ability of MGNs *in vivo* by comparing to control GNs (*n* = 5) conjugated with isotype-matched anti-IgG and Raman reporters (*n* = 5) (GNs/anti-IgG/pMBA + GNs/anti-IgG/DTNB at a 1 : 1 ratio). (e) *In vivo* SERS spectra at 6 h post injection of control IgG GNs (black) and MGNs (orange) groups. Longitudinal signal throughout tumor between the two groups tracked for (f) pMBA signal at 1076 cm^−1^ (conjugated with anti-VEGFR2 or anti-IgG) (g) DTNB signal at 1331 cm^−1^ (conjugated with anti-CD8 or anti-IgG). Statistical significance is reported by a 2-tailed student's *t*-test (*p* ≥ 0.05: ns., *p* < 0.05: *, *p* < 0.01: **, *p* <0.001: ***).

Next, we examined if multiplexed gold nanostars (MGNs) have high accumulation in the TME and enable targeted detection of both VEGFR2^+^ tumor cells and CD8^+^ T cells in untreated 4T1 murine tumors (Fig. 2a). The dose of MGNs delivered for SERS imaging impacts the overall SERS signal in tumors where too high of a dose can induce agglomeration of nanoparticles and dampen the SERS signal. A dose-dependent study was conducted where mice in the high-dose cohort received 200 µL of 6 mg mL^−1^ MGNs (1.2 mg total per mouse), while the low-dose cohort was administered 200 µL of 3 mg mL^−1^ GN (0.6 mg total per mouse) consistent with earlier protocols (Fig. 2b–d).^27,34^ Prior to MGNs administration, SERS imaging was performed to establish a baseline (0 h) spectral profile of the tumors in each mouse. MGNs were administered *via* intraperitoneal (IP) injection, a delivery route that has been successful for long systemic circulation of GNs and for accumulation in subcutaneous tumors.^23,27^ Based on prior studies from our group, longitudinal accumulation of MGNs peak at 6 h post-administration, followed by a marked decline in signal intensity by 42 h.^27^ Thus, for this study, SERS imaging was performed at 6 h, 20 h, and 50 h post-injection to track longitudinal changes in signal. Acquired spectra were normalized to the 1448 cm^−1^ band representative of intrinsic tissue components such as proteins and lipids. Additional normalization was performed relative to the baseline (0 h) signal intensity for the target-specific Raman peaks: 1076 cm^−1^ for GNs/anti-VEGFR2/pMBA and 1331 cm^−1^ for GNs/anti-CD8/DTNB. Average spectral intensities were calculated for each cohort to allow longitudinal comparisons across timepoints. Notably, mice that received the high-dose of MGNs had significantly dampened SERS signal *in vivo* relative to the mice that received the low-dose of MGNs (Fig. 2b–d), which may reflect nanoparticle aggregation at higher concentrations. Based on these observations, the lower MGNs dose was selected for all subsequent experiments. At the high MGNs dose, we also observed substantial nanoparticle aggregation by the 50 h timepoint. While a moderate degree of aggregation can enhance SERS performance, the extent of aggregation at this timepoint resulted in signal dampening. Consequently, the signal-to-noise ratio (SNR) at 50 h was insufficient for reliable quantification, and therefore, the 50 h data was excluded from the analysis. We also conducted computed tomography (CT) imaging of these mice (SI Fig. S4) since gold nanoparticles (AuNPs) have served as CT contrast agents.^35^ But exceptionally high doses of AuNPs are typically required to achieve high SNR in CT. Our results indicate the low dose of MGNs that was sufficient to acquire strong SERS signal *in vivo* was insufficient for high CT contrast *in vivo*. This suggests the potential of SERS as a clinically relevant imaging approach that can surpass the challenges of current clinical techniques.

Next, we confirmed the specificity of MGNs in targeted detection of both immune cells and tumor cells *in vivo* by comparing them to GNs that were conjugated with isotype-matched nonspecific IgG antibodies (Fig. 2e–g). One group of mice received MGNs (GNs/anti-VEGFR2/pMBA + GNs/anti-CD8/DTNB at a 1 : 1 ratio) and the other group received control IgG GNs (GNs/anti-IgG/pMBA + GNs/anti-IgG/DTNB at a 1 : 1 ratio) at the same low dose indicated earlier (0.6 mg). *In vivo* SERS imaging showed the strongest signal at 6 h post-injection, with significantly higher intensities observed for MGNs cohort compared to the control IgG GNs cohort up to 20 h post *in vivo* delivery. This suggests that MGNs actively target specific cells in the tumors *via* targeting mAbs and there is limited passive accumulation within tumors. The high *in vivo* SERS signal at 6 h timepoint was primarily attributed to GNs localization at the tumor periphery. Over time as GNs redistributed deeper into tumors, a corresponding decline in SERS signal was observed attributed to depth limitations of RS. This trend was consistent across both cohorts and in line with previous observations (Fig. 2b, c, e–f).^27,36^

To ensure reproducibility and consistency in *in vivo* SERS imaging, several control measures were implemented as follows:

(1) Hair on mice flank was removed using a depilatory cream (Nair Sensitive Bikini Cream Hair Remover) prior to imaging at both the 0 h and 24 h timepoints to eliminate interference as the presence of hair can oversaturate the SERS signal.

(2) SERS imaging was performed in high confocal mode with the focal point offset −100 µm below the surface of the tumor, which was crucial in obtaining high SNR. We observed that imaging the tumor surface, whether in a “standard” or high confocal mode, resulted in a high tissue background signal. Offsetting the focal point below the surface essentially minimized surface-level tissue background to enable high SNR of accumulated nanoparticles.

(3) Further, spectra were acquired at 0 h (pre-injection) for each mouse that served as internal background reference allowing normalization of spectral intensities at subsequent timepoints to each individual mouse's baseline. This approach minimized inter-mouse variability and allowed accurate comparison across groups.

(4) Consistent batch-to-batch reproducibility of MGNs synthesis was ensured by performing the synthesis under standardized conditions. HEPES buffer (0.2 M) was freshly prepared in Milli-Q water (initial pH: 5.4–5.6), adjusted to pH 7.41 ± 0.02. Every new batch of HEPES is screened prior to use, as not all commercial batches yield reproducible gold nanostars (GNs) growth. Screening is done by comparing the extinction intensity using UV-Vis, and the average size diameter using DLS with the previous data. Once a consistent batch is identified, multiple bottles are ordered from the same lot number to preserve batch-to-batch uniformity. Gold chloride stock (nominal 100 mM) was prepared in Milli-Q water and stored at 4 °C for up to 6–8 months. However, the actual working concentration was determined empirically by screening new gold stocks against prior validated stocks using UV-Vis absorbance spectra, DLS, and zeta potential measurements.

(5) Each MGNs batch was characterized by UV-Vis spectroscopy to confirm that the longitudinal surface plasmon resonance (LSPR) peak as shown in Fig. 1b and c. For Bare GNs it was within 760–775 nm, for pMBA/DTNB labeled GNs we observed a 12–15 nm shift and for the final MGNs solution we observed a final shift of 30–40 nm compared to the Bare GNs. Further, the full width at half maximum (FWHM) from the extinction plot, as shown in Fig. 1h, was observed to be between 225 ± 9 nm for pMBA-labeled GNs and 235 ± 15 nm for DTNB-labeled GNs. As synthesized bare GNs had a hydrodynamic diameter of 35.0 ± 1.7 nm and zeta potential of −38.5 ± 1.1 mV. pMBA-labeled GNs measured 41.5 ± 2.0 nm (−33.2 ± 0.7 mV), and DTNB-labeled GNs measured 40.6 ± 2.5 nm (−31.7 ± 1.4 mV). We aimed for these sizes to maintain batch-to-batch reproducibility.

(6) Raman system calibration was performed daily using an external silicon standard, with the 520.4–520.6 cm^−1^ peak constrained to 68 000–70 000 counts. All Raman spectra were baselined in WiRE software, and the freshly synthesized batch of GNs was expected to fall within ±10% of 2000 counts for pMBA-labeled GNs (1076 cm^−1^) and ±10% of 3900 counts for DTNB-labeled GNs (1331 cm^−1^). Only batches meeting this calibration threshold were used for imaging.

**Fig. 3:**
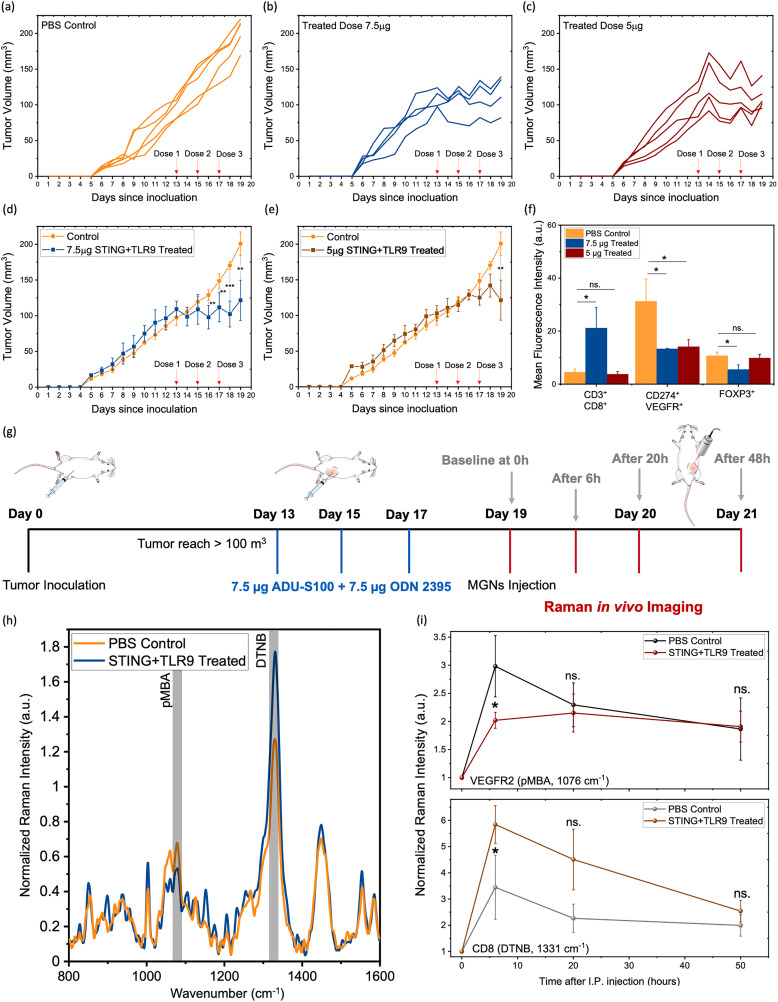
Treatment response measured with multiplexed gold nanostars (MGNs) in 4T1 tumor model in response to STING + TLR9 immunotherapy. Individual tumor volumes of mice (a) in the PBS control cohort (orange, *n* = 5) (b) and (c) and dose-dependent STING + TLR9 treatment group compared to (b) 7.5 µg treated group (blue, *n* = 5), and (c) 5 µg treated group (brown, *n* = 5). (d) and (e) Average tumor volume (d) compared between PBS control, and 7.5 µg treated groups (e) compared between PBS control, and 5 µg treated groups. (f) Flow cytometry analysis of CD8^+^ T cells (CD3^+^ CD4^−^ CD8^+^), VEGFR2 (CD274^+^ VEGFR2^+^) tumor cells and FOXP3^+^*T*_reg_ cells (FOXP3^+^ CD25^+^) in PBS control and the two doses of STING + TLR9 treated groups. (g) Schema of immunotherapy regimen, MGNs delivery, and SERS imaging *in vivo* in 4T1 tumor model. (h) SERS spectra at 6 h post injection of PBS control mice (orange, *n* = 5) and STING + TLR9 treated at 7.5 µg mice (blue, *n* = 5) and received 0.6 mg of MGNs (GNs/anti-VEGFR2/pMBA + GNs/anti-CD8/DTNB at a 1 : 1 ratio). (i) Longitudinal tracking of VEGFR2 (*via* pMBA peak) expression and CD8 (*via* DTNB peak) expression between the cohorts. Statistical significance is reported by a 2-tailed student's *t*-test (*p* ≥ 0.05: ns., *p* < 0.05: *, *p* < 0.01: **, *p* <0.001: ***).

Next, we examined if SERS *in vivo* with multiplexed gold nanostars (MGNs) can track CD8^+^ T cells and VEGFR2^+^ tumor cells in response to STING + TLR9 agonist immunotherapy. First, we conducted a dosing study to determine the ideal dose of immunotherapy to assess a therapeutic response. This included (i) 7.5 µg each of ADU-S100 (STING agonist) and ODN2395 (TLR9 agonist), and (ii) 5 µg of each agonist; for both doses, the treatment was administered in a total volume of 30 µL with a concentration of 0.5 mg mL^−1^ of ADU-S100 in DPBS, and 0.5 mg mL^−1^ of ODN2395 in endotoxin-free water. Mice were randomized into three experimental cohorts and subcutaneously inoculated with 0.5 million 4T1 tumor cells. Treatment commenced once tumors reached an average volume between 100–120 mm^3^. Intratumoral (IT) injections were performed on alternate days for a total of three administrations. IT injection is the preferred delivery method for STING and TLR9 agonists due to their rapid degradation *in vivo* if delivered systemically.^14,15^ Control animals received PBS injections (30 µL) on a staggered schedule (days 13, 15, and 17). At the end of the study, mice were euthanized, and the tumors were harvested and homogenized into single-cell suspensions for FACs. Tumor volume measurements indicated a significant therapeutic benefit following immunotherapy, with statistically significant differences observed for both treatment cohorts, but a higher therapeutic response was achieved for mice that received 7.5 µg of each agonist (Fig. 3a–e). Average tumor weights also showed a significant difference between the cohorts (SI Fig. S5a and b). Flow cytometry analysis of tumors indicated that the 7.5 µg dose of each agonist induces a statistically significant increase in cytotoxic CD8^+^ T cells (CD3^+^ CD4^−^ CD8^+^), a significant decrease in VEGFR2 tumor cells (CD274^+^ VEGFR2^+^) as well as a decrease in FOXP3^+^ regulatory *T* cells (*T*_reg_) (FOXP3^+^CD25^+^) and CD4^+^ T cells (CD3^+^ CD4^+^ CD8^−^) (Fig. 3f; SI Fig. S5c). These results indicate that the higher dose of immunotherapy elicits robust activation of cytotoxic immune responses, suppresses angiogenic signaling and pro-tumor *T*_regs_, and improves dendritic cell function and IFN-1 signaling, thus inducing adaptive antitumor immunity.^15,37,38^ The FACS gating strategy is provided in SI Fig. S6 and S7. Based on this dosing study, the 7.5 µg dose of both agonists was selected as the optimal IT regimen for SERS imaging *in vivo*.

The utility of SERS imaging allows us to examine treatment response to immunotherapies *in vivo* in real time. Immunotherapy dosing was congruent to that for the FACS study described above. The day after the third dose of treatment, baseline tissue *in vivo* SERS spectra were taken for each tumor necessary for quantitative analysis. Two days following the last treatment dose, 0.6 mg of MGNs (GNs/anti-VEGFR2/pMBA + GNs/anti-CD8/DTNB at a 1 : 1 ratio) were IP delivered in mice, and SERS imaging was conducted *in vivo* (Fig. 3g). Changes in SERS spectral intensities of pMBA at 1076 cm^−1^ and DTNB at 1331 cm^−1^ indicated changes in VEGFR2^+^ and CD8^+^ expression respectively for the STING + TLR9-treated cohort (Fig. 3h). Quantitative longitudinal analysis of SERS data indicated a significant reduction in VEGFR2^+^ expression and a significant increase in CD8^+^ T cell infiltration as early as 6 h post MGNs injection in the immunotherapy cohort (Fig. 3i). These findings show that downregulation of VEGFR2 and enrichment of CD8^+^ T cells post immunotherapy can be straightforwardly imaged *in vivo* with SERS enabling simultaneous tracking of multiple markers with multiplexed MGNs. These results correspond well with trends observed in tumor volumes (Fig. 3a–e) and flow cytometry analysis (Fig. 3f) and align with literature findings that indicate STING + TLR9 agonists normalize tumor vasculature and enhance immune cell infiltration.^32^

**Fig. 4:**
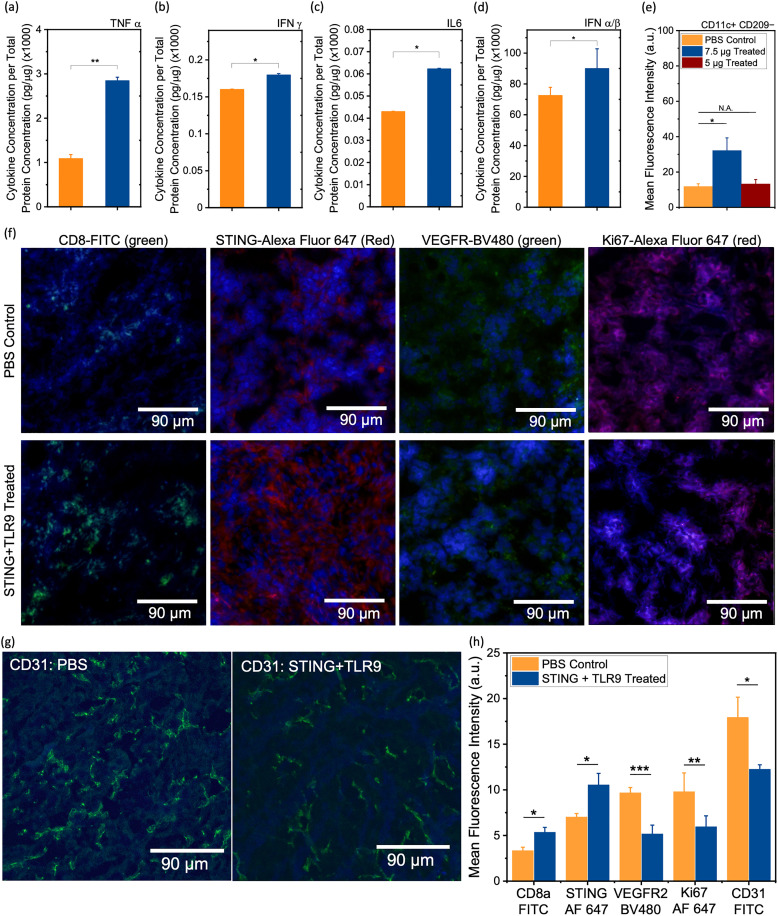
*Ex vivo* Quantification of Immune Profile in Tumors treated with STING + TLR9. (a)–(d) Serum cytokine concentrations by ELISA for PBS control (orange, *n* = 5) and 7.5 µg STING + TLR9 (blue, *n* = 5) mice for (a) TNF-α, (b) IFN-γ, (c) IL-6, and (d) systemic STING activation *via* IFN-α. (e) Flow cytometry analysis of CD11c^+^ CD209^−^ dendritic cells in tumors. (f) Representative 20× immunofluorescence images for CD8a (FITC), STING (Alexa Fluor 647), VEGFR (BV480) and Ki67 (Alexa Fluor 647) in PBS control and STING + TLR9 immunotherapy treated tumors. (g) Representative 20× immunofluorescence images for (CD31) of the PBS control, and STING + TLR9 combination immunotherapy cohorts. (h) Quantification of IF signals for CD8, STING, VEGFR2, Ki67 and CD31 in tumor sections of PBS Control (*n* = 3) and STING + TLR9 treated (*n* = 3) tumors. Corresponding raw, unprocessed IF images are provided in the (SI Fig. S8) for reference. Statistical significance is reported by a 2-tailed student's *t*-test (*p* ≥ 0.05: ns., *p* < 0.05: *, *p* < 0.01: **, *p* <0.001: ***).

Treatment response to immunotherapies *in vivo* was next validated with pre-clinical assays including cytokine analysis in mice sera with enzyme-linked immunosorbent assays (ELISA) for TNF-α, IFN-γ, and IL-6 and the systemic IFN-1 response was examined with IFN-α/β (Fig. 4a–d). Proinflammatory cytokines are key mediators of antitumor immunity indicating systemic innate and adaptive immune activation. A significant elevation of these cytokines was observed in the STING + TLR9 treated cohort relative to PBS control. STING activation promotes IFN-1 signaling and enhances dendritic cell-mediated cross-priming of CD8^+^ T cells, leading to increased IFN-γ production,^39^ while TLR9 activation by CpG DNA induces TNF-α and IL-6 *via* MyD88-dependent NF-κB signaling.^40^ Additionally, the elevated levels of IFN-α/β observed in serum are attributed to TLR9-mediated activation of plasmacytoid dendritic cells, which produce high levels of IFN-1 through MyD88-dependent IRF7 signaling.^41,42^ This systemic increase in IFN-α/β further supports the immune activation induced by the combination treatment. We also examined CD11c^+^ CD209a^−^ DC activation (Fig. 4e) for both doses of the STING + TLR9 treatment. Given that CD209a (DC-SIGN) is associated with immature and tolerogenic DC subsets in mice, the observed increase in CD209a^−^ DCs in the 7.5 µg dosage cohort suggests functional maturation within the TME as an effect of innate immune stimulation. This is consistent with previous studies showing an upregulation of DC activation markers following STING and TLR9 activation.^37^ Next, immunofluorescence (IF) imaging of tissues for expression of CD8, STING, VEGFR2, Ki67 proliferation marker and CD31 was conducted (Fig. 4f and g) and quantified (Fig. 4h). Our results showed a significant increase in CD8^+^ T cell recruitment, STING activation, and significant decrease in VEGFR2, Ki67 and CD31 expression in the STING + TLR9 immunotherapy cohort relative to PBS control. Previous studies have shown a positive correlation between VEGFR2 and proliferation as measured by Ki67.^43^ Furthermore, IF imaging of tissues for the expression of CD31,^44^ showed a significant decrease in the STING + TLR9 immunotherapy cohort relative to the PBS control cohort. These findings collectively suggest the role of VEGFR2 in supporting tumor cell proliferation and angiogenesis. These observations validate our *in vivo* SERS results of an increase in CD8 and decrease in VEGFR levels in response to treatment. Further, hematoxylin and eosin (H&E) staining of tumor tissues (SI Fig. S5) showed increased disruption of tumor vasculature and stromal architecture in the treated cohort as compared to the PBS control cohort, which is corroborated by previous studies.^45^

**Fig. 5:**
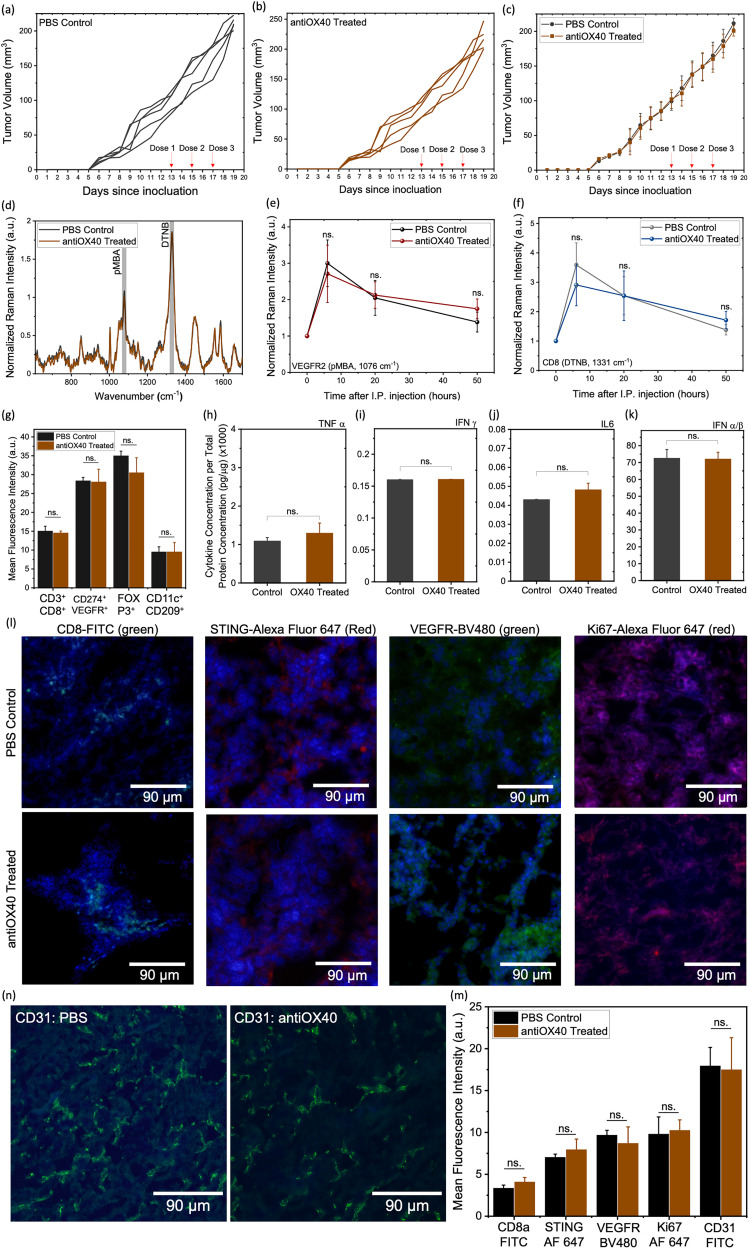
Treatment resistance measured with multiplexed gold nanostars (MGNs) in 4T1 tumor model in response to antiOX40 immunotherapy. (a) and (b) Individual tumor volumes for (a) PBS control (black, *n* = 5) and (b) antiOX40 treated (brown, *n* = 5) cohorts. (c) Group average tumor volumes comparing PBS control and antiOX40 treatment. (d) Representative SERS spectra acquired 6 h post-injection from PBS control (black, *n* = 5) and antiOX40 (brown, *n* = 5) mice following administration of 0.6 mg MGNs (GNs/anti-VEGFR2/pMBA plus GNs/anti-CD8/DTNB at a 1 : 1 ratio). (e) and (f) Longitudinal tracking of (e) VEGFR2 (*via* pMBA peak) expression (f) CD8 (*via* DTNB peak) expression between the groups. (g) Flow cytometry analysis of CD8^+^ T cells (CD3^+^ CD4^−^ CD8^+^), VEGFR2 (CD274^+^ VEGFR2^+^) tumor cells and FOXP3^+^*T*_reg_ cells (FOXP3^+^ CD25^+^) in PBS control *versus* antiOX40 treated cohorts. (h)–(k) Serum cytokine concentrations by ELISA for PBS control (black, *n* = 5) and antiOX40 treated (brown, *n* = 5) mice, (h) TNF-α, (i) IFN-γ, (j) IL6, and (k) systemic STING activation *via* IFN-α. (l) Representative 20× IF images for CD8a (FITC), STING (Alexa Fluor 647), VEGFR (BV480) and Ki67 (Alexa Fluor 647) in PBS control and antiOX40 treated tumors. (m) Quantification of IF signals for CD8, STING, VEGFR2, Ki67 and CD31 in tumor sections of PBS Control (*n* = 3) and antiOX40 treated (*n* = 3) tumors. (*n*) Representative 20× immunofluorescence images for (CD31) of the PBS control, and antiOX40 monotherapy cohorts. Corresponding raw, unprocessed IF images are provided in the (SI Fig. S10) for reference. Statistical significance is reported by a 2-tailed student's *t*-test (*p* ≥ 0.05: ns., *p* < 0.05: *, *p* < 0.01: **, *p* <0.001: ***).

We next examined multiplexed gold nanostars (MGNs) ability to differentiate resistance to immunotherapy with *in vivo* SERS in the 4T1 tumor model that is resistant to antiOX40 immunotherapy. Blocking OX40 receptors represents a promising therapeutic strategy in BC to promote effector CD4^+^/CD8^+^ responses and attenuate *T*_reg_-mediated suppression.^46^ However, in TNBC, antiOX40 monotherapy has limited efficacy due to its aggressive growth kinetics, poor immunogenicity, and immunosuppressive TME that limits T-cell infiltration and function.^47^ Therefore, we chose antiOX40 in our 4T1 model to demonstrate SERS can distinguish nonresponders of treatment. Mice received antiOX40 *via* IT delivery at the same dosage used in the STING + TLR9 cohort. For SERS imaging *in vivo*, mice received an identical dosage of MGNs *via* IP delivery. As expected, 4T1 tumors were resistant to antiOX40 as seen from tumor growth curves with no statistically significant difference observed between the treatment and PBS control cohorts (Fig. 5a–c), and average tumor weights also showed no significant difference (SI Fig. S9a and b). Consistent with this outcome, SERS spectral profiles showed minimal variation across treatment arms (Fig. 5d). Longitudinal monitoring of VEGFR2 using pMBA-tagged MGNs (Fig. 5e) and CD8^+^ T cells *via* DTNB-conjugated MGNs (Fig. 5f) revealed negligible changes between the two cohorts. *In vivo* endpoints were validated with *ex vivo* assays including flow cytometry analysis of immune cell populations. Our data showed no significant differences in the recruitment of CD8^+^ T cells (CD3^+^ CD4^−^ CD8^+^), VEGFR2^+^ tumor cells (CD274^+^ VEGFR2^+^), FOXP3^+^ regulatory T cells (*T*_reg_) (FOXP3^+^ CD25^+^), CD4^+^ T cells (CD3^+^ CD4^+^ CD8^−^) or mature DC (CD11c^+^ CD209a^−^) between the two cohorts as well (Fig. 5g and SI Fig. S9c). Cytokine analysis also showed minimal differences between the treatment and PBS control cohorts (Fig. 5h–j) and no difference was observed between the cohorts for IFN-α (Fig. 5k) suggesting the IFN-1 response is not elicited with antiOX40 treatment in the 4T1 tumor model. Furthermore, IF imaging of tissues for expression and quantification of CD8, STING, VEGFR2, Ki67 and CD31 (Fig. 5l–n) did not show a significant difference between the control and antiOX40 treatment. The H&E images (SI Fig. S9d) corroborated these findings. Collectively, these results support our SERS *in vivo* imaging results indicating that MGNs can effectively distinguish between responders and nonresponders of immunotherapies.

In addition to *in vivo* SERS spectral imaging, we also examined *ex vivo* SERS maps of tumor sections to enable a spatial map of CD8^+^ and VEGFR2^+^ biomarker distribution which cannot be captured by *in vivo* imaging. Such spatial context of biomarker distribution enables high resolution (at a cellular level) molecular makeup of the tumors and an understanding of the cellular heterogeneity in cancer. *Ex vivo* SERS maps of tumors surpass many of the challenges of conventional approaches such as IF. These include time- and labor-intensive procedures, several processing steps including antigen retrieval, use of multiple labels to enable multiplexing, optimization required for each label and multiple wash steps to remove excess label, and a limited window to accomplish the imaging due to rapid photobleaching of fluorophores. Further, tissue autofluorescence often compromises the reliability and accuracy of the final fluorescence maps. Our SERS maps of tumor sections allowed for high spatial resolution and multiplexed profiling of both CD8^+^ and VEGFR2^+^ without any of the cumbersome steps involved in IF as the distribution of multiplexed gold nanostars (MGNs) (following *in vivo* delivery) are directly captured in tissues. In addition, there are no limitations of photobleaching, and well-established approaches now exist that allow us to easily remove tissue background from SERS data.^26,48^ Our goal is not to replace IF but complement it with SERS to enable comprehensive spatial profiling of the TME.

**Fig. 6:**
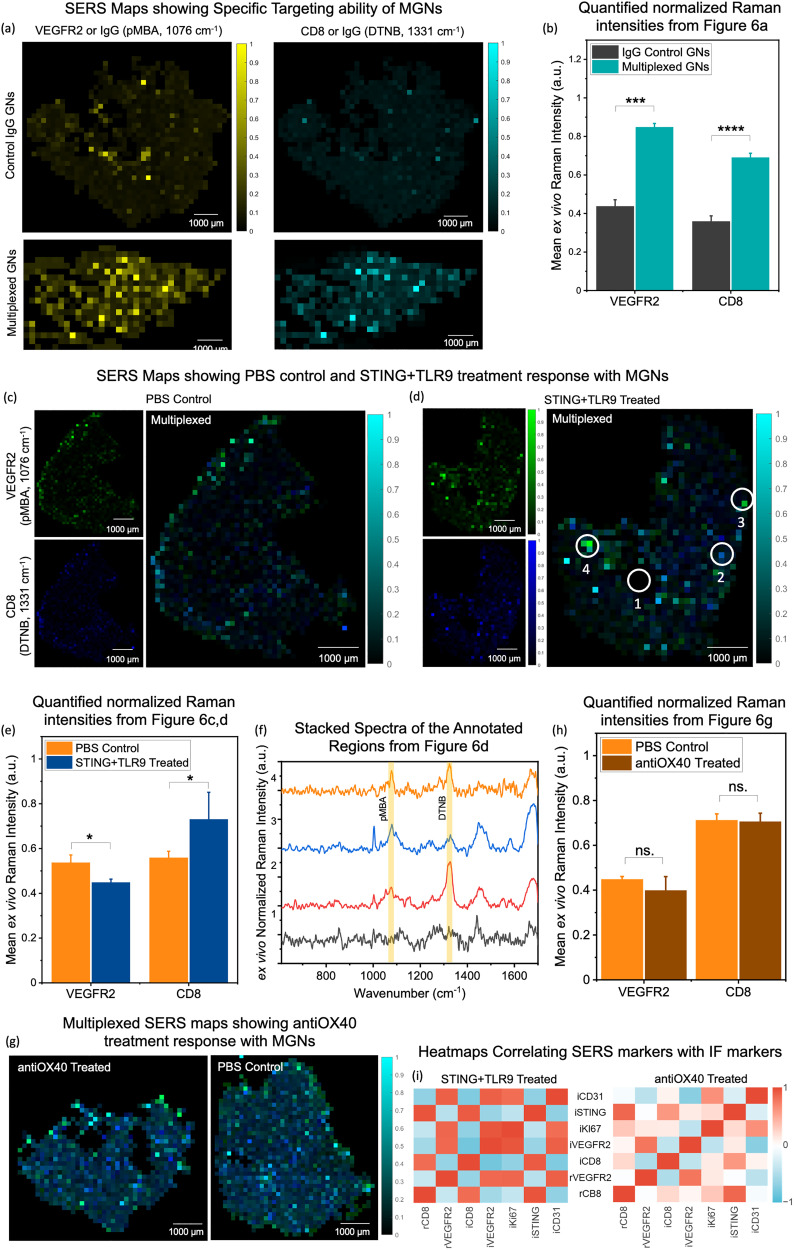
*Ex vivo* SERS spatial maps of tumors and correlation to immunofluorescence markers. (a) and (b) Representative *ex vivo* SERS maps acquired over an entire tumor section (200 µm per pixel) for the IgG control GNs and targeted MGNs, showing signal from VEGFR2 or IgG (pMBA, 1076 cm^−1^; yellow) and CD8 or IgG (DTNB, 1331 cm^−1^; cyan), normalized to the 1448 cm^−1^ lipid band. (b) Quantified mean intensities of VEGFR2 or IgG (pMBA, 1076 cm^−1^) and CD8 or IgG (DTNB, 1331 cm^−1^) comparing control IgG GNs (black, *n* = 3) and MGNs (blue, *n* = 3). (c) and (d) Representative *ex vivo* SERS maps for (c) PBS control and (d) STING + TLR9–treated tumors, displayed as single channels, VEGFR2 (pMBA, 1076 cm^−1^; green) and CD8 (DTNB, 1331 cm^−1^; blue) and as multiplexed overlays. (e) Quantified mean intensities of VEGFR2 (pMBA, 1076 cm^−1^) and CD8 (DTNB, 1331 cm^−1^) for PBS control (orange, *n* = 3) and STING + TLR9 (blue, *n* = 3). (f) Stacked spectra extracted from four annotated regions of the STING + TLR9 multiplexed representative map: Regions of interest (ROI) shown include ROI-1, background (black, no detectable pMBA or DTNB features); ROI-2, CD8-DTNB positive (red, pronounced 1331 cm^−1^ band); ROI-3, VEGFR2-pMBA positive (blue, pronounced 1076 cm^−1^ band); ROI-4, multiplexed region (orange, concurrent 1331 cm^−1^ and 1076 cm^−1^ features). Spectra are baseline-corrected and normalized for display. (g) Representative *ex vivo* SERS maps showing multiplexed signal intensity of VEGFR2 (pMBA, 1076 cm^−1^; green) and CD8 (DTNB, 1331 cm^−1^; blue) of antiOX40 treated and PBS control cohorts. (h) Quantified mean intensities of VEGFR2 (pMBA, 1076 cm^−1^) and CD8 (DTNB, 1331 cm^−1^) comparing PBS Control (orange, *n* = 3) and antiOX40 treated (brown, *n* = 3) cohorts. (i) Pearson's correlation heatmaps showing association of marker pairs quantified by *ex vivo* SERS and IF imaging for STING + TLR9 (*n* = 3) and antiOX40 (*n* = 3) treated cohorts. The prefix “r” indicates *ex vivo* SERS measurements, and “i” indicates IF measurements. Statistical significance is reported by a 2-tailed student's *t*-test (*p* ≥ 0.05: ns., *p* < 0.05: *, *p* < 0.01: **, *p* <0.001: ***).

Here, following *in vivo* imaging, tumors were harvested 72 h post-injection of MGNs and flash frozen. Cryo-preserved tumors were sectioned at a thickness of 8 µm, placed on calcium fluoride (CaF_2_) slides to minimize background SERS interference, and SERS maps were acquired with a 785 nm laser with a 200 µm × 200 µm resolution step size. A custom MATLAB pipeline was used to subtract tissue autofluorescence, normalize signal intensities across samples, and extract the peaks corresponding to each Raman label. The resulting maps were color-coded by each label providing spatial representations of the distribution of GNs/anti-VEGFR2/pMBA and GNs/anti-CD8/DTNB in the tissue. We first examined the *ex vivo* SERS maps of tumors collected from the study shown in Fig. 2 where we compared multiplexed GNs (GNs/anti-VEGFR2/pMBA and GNs/anti-CD8/DTNB) relative to control IgG GNs (GNs/anti-IgG/pMBA + GNs/anti-IgG/DTNB) (Fig. 6a). These SERS maps, corresponding to actively targeted *vs.* passive accumulation in tumors, provide a measurable validation of the *in vivo* results seen in Fig. 2 where significantly higher intensities are observed in the targeted MGNs groups relative to control IgG GNs, as quantified in Fig. 6b. These SERS maps also indicated that MGNs are distributed throughout the tumor, not just the periphery or tumor core. Next, SERS maps were compared between the STING + TLR9 treated and PBS control groups (Fig. 6c and d) and the quantification of peak intensities of VEGFR2 (pMBA, 1076 cm^−1^) and CD8 (DTNB, 1331 cm^−1^) (Fig. 6e) show a significant difference between control and treatment. *Ex vivo* SERS maps of tumor sections with MGNs resolved discrete spatial domains and their reporter signatures. Our goal in this comparison was to examine spatially resolved visualization of multiplexed distribution of CD8^+^ and VEGFR2^+^ in tumors in the responders *vs.* nonresponders and validation of *in vivo* SERS endpoints as seen in Fig. 3 and 5. The maps revealed the distribution of VEGFR2+ tumor cells (green, pMBA 1076 cm^−1^) and CD8^+^ T cells (blue, DTNB 1331 cm^−1^) throughout the tumor, which corresponded well with the IF images shown in Fig. 4 and 5.

The multiplexed SERS map, where a mix of green and blue yields cyan, shows tumor sections that have (i) a higher VEGFR2^+^ distribution (bright green), (ii) higher CD8^+^ distribution (bright dark blue), (iii) where both biomarkers are present equally (bright cyan) or at various ratios (various shades of cyan) and (iv) where neither marker is at detectable levels (near black). Representative regions of interest (ROIs) were selected to illustrate these distinct spectral features (Fig. 6f), ROI 1 defined a local background with no detectable reporter features; ROI 2 exhibited a dominant DTNB signal at 1331 cm^−1^ with minimal pMBA contribution, indicating CD8^+^ T cell enrichment; ROI 3 showed a dominant pMBA signal at 1076 cm^−1^ with minimal DTNB, indicating VEGFR2 distribution; and ROI 4 displayed concurrent 1076 and 1331 cm^−1^, indicating presence of both CD8 and VEGFR2 markers at the measured spatial resolution. The spatial distribution of these distinct markers underscores the utility of multiplexed SERS mapping in resolving the localization of distinct molecular targets with high precision. The nonresponder treatment group (antiOX40 treated *vs.* PBS control) (Fig. 6g) were also compared and the quantification (Fig. 6h) shows no significant differences between PBS control (orange) and antiOX40 (brown) treated cohorts. Quantitative analysis of the SERS maps corresponds well with *in vivo* SERS imaging results.

To understand how the various biomarkers we measured in tissues with IF (iCD8, iVEGFR2, iSTING, iCD31, iKi67) correspond to the markers measured with *ex vivo* SERS maps (rCD8, rVEGFR2), we generated cross-modal Pearson's correlation heatmaps incorporating SERS and IF markers (Fig. 6i). The prefix “r” indicates *ex vivo* SERS measurements, and “i” indicates IF measurements. In the heatmaps, blue cells denote an inverse correlation, and red cells indicate a direct correlation; *r* values between ± (0–0.32) suggest a weak correlation, ± (0.33–0.65) indicate a moderate correlation, and ± (0.66–1.00) denote a strong correlation of variates to each other. The correlation ratios for this heatmap are available in the (SI Table S2). In the STING + TLR9 cohort, we observed moderate to strong correlations. As expected, rCD8 correlated strongly with iCD8 (*r* = 0.84), and rVEGFR2 with iVEGFR2 (*r* = 0.86), supporting concordance between *in vivo* SERS and IF endpoints and consistent with *in vivo* results (Fig. 3 and 5). Proliferation marker measurements were aligned with angiogenic signaling where iKi67 positively correlated strongly with rVEGFR2 (*r* = 0.87) and iVEGFR2 (*r* = 0.95). iKi67 inversely correlated to T-cell markers showing moderate association to rCD8 (*r* = −0.38) and strong correlation to iCD8 (*r* = −0.67). iSTING correlated strongly with both rCD8 (*r* = 0.96) and iCD8 (*r* = 0.93), consistent with our *in vivo* findings and other studies that support STING activation enhancing CD8^+^ T-cell recruitment.^19^ iSTING was inversely associated with rVEGFR2 (*r* = −0.45) and iVEGFR2 (*r* = −0.63). These trends support STING-driven vascular normalization and attenuation of angiogenic programs,where effects were not observed in STING-deficient mice upon agonist treatment.^32,49,50^ We also find that iCD31 was inversely correlated with iSTING (*r* = −0.68) but jointly correlated with rVEGFR2 (*r* = 0.90) and iVEGFR2 (*r* = 0.90), indicating that STING activation and therapeutic response reduces vascular density and angiogenic signaling.

In contrast, correlations in the antiOX40 monotherapy group were predominantly weak, with only occasional moderate or strong relationships. Notably, iSTING and iCD31 were essentially uncorrelated (*r* = −0.05). A likely interpretation is that STING protein is innately detectable but insufficiently activated in 4T1, where cGAS–STING signaling is dampened. Since antiOX40 does not directly activate this pathway, the vascular normalization as observed with STING agonist treatment would not be expected under antiOX40 alone, thus iSTING shows little dependence on CD31. This is consistent with the limited biological activity of OX40 monotherapy in 4T1 relative to combination regimens.^51^

## Conclusion

In conclusion, this work leverages the ability of multiplexed gold nanostars (MGNs) to accumulate in tumors and enable accurate and highly sensitive multiplexed profiling of lymphocytes and cancer cells in real time *in vivo*. Our findings show that MGNs simultaneously track CD8^+^ T cells and VEGFR2^+^ expressing tumor cells in 4T1 tumors *in vivo* with multiplexed SERS imaging, enabling us to predict treatment response to STING + TLR9 immunotherapy. Further, MGNs also distinguished nonresponders of treatment in 4T1 tumors treated with antiOX40 therapy. *In vivo* endpoints were validated with *ex vivo* tissue analysis. Our results showed STING + TLR9 treatment in 4T1 tumors increased T cell recruitment and decreased VEGFR2 levels while no change in these markers was observed in nonresponder antiOX40 therapy. Further, *ex vivo* SERS maps of tumors provided the spatial distribution of distinct cell types in the tumor. Our findings indicate that our approach may ultimately enable a clinically translatable system to measure spatial heterogeneities at the single cell level that are often missed in bulk tissue measurements. A correlation analysis of *ex vivo* SERS endpoints to markers measured with IF showed moderate to strong association in tumors that were responsive to treatment and weak correlation to tumors that were resistant. These results reinforce that independent imaging modalities converge on the same biological trends. Further, our findings also validate that MGNs mediated SERS has the potential to compliment and surpass the limitations of time- and labor-intensive IF. Whereas our results are encouraging, several challenges remain in translating MGNs and SERS imaging into clinical practice. Nanoparticle-based contrast agents require rigorous regulatory evaluation for safety, reproducibility, and biocompatibility, particularly for repeated systemic administration. Ensuring GMP-compliant, scalable synthesis of MGNs with consistent physiochemical and targeting properties is also essential. Emerging advances in nanoparticle manufacturing through state-of-the-art facilities are expected to address scalability. While Raman instrumentation is currently not widely accessible in clinical settings, the recent adoption of Raman spectroscopy in Veterans Affairs Boston and Tampa healthcare systems supports its clinical relevance and that efforts are ongoing for wide accessibility.^52^ Since gold nanoparticles have been used in human studies, and Raman spectroscopy is now being tested in patients across multiple clinical trials (*e.g.*, NCT00060580, NCT06384924), we envision that most of these regulatory hurdles are addressable.

We expect that in the future multiplexed SERS mapping integrated with spatial transcriptomics and immunohistochemistry may enable a multimodal immune atlas of the TME. Future studies should prioritize expansion of the Raman reporter panel that are stable *in vivo* to allow real time tracking of a milieu of biomarkers in tumors enabling a deeper biological interpretation of the interplay of various cell types in the TME.

## Materials and methods

### Synthesis and characterization of SERS nanoprobes

The synthesis of gold nanoprobes was performed over several days. Targeting antibodies, anti-CD8a (Bio X Cell, clone: 53–6.7) and anti-VEGFR2 (Bio X Cell, clone: DC101) were first prepared individually at a concentration of 1 mg mL^−1^ in 0.1 M sodium bicarbonate. The *ortho*-pyridyl disulfide polyethylene glycol succinimidyl valerate (OPSS-PEG-SVA) linker (Laysan Biosciences, 2000 kDa) was also prepared at 69 mg mL^−1^ in sodium bicarbonate and combined with the antibody solutions at a mole ratio of 1 : 500 and a volume ratio of 9 : 1 antibody to linker solution. The mixture was incubated on an inverting rotator at 4 °C for 22–24 h. On the following day, gold nanostars were synthesized in batches of 30 mL through an unseeded process. Gold(iii) chloride (Sigma) was prepared at a stock concentration of 100 mM in milliQ water and stored at 4 °C. The HEPES buffer (Fisher) was prepared at 0.2 M in milliQ water, adjusted to pH 7.4 (±0.02) using 1 M sodium hydroxide (Fisher), and mixed gently. For a single batch, 12 mL of 0.2 M HEPES was combined with 18 mL of milliQ water in a 50 mL falcon tube and mixed by gentle inversion up to 30 times. Subsequently, 300 µL of 16.67 mM gold chloride trihydrate was added to the HEPES solution in a darkened room and mixed by gentle inversion for 50 cycles. The solution was then incubated at room temperature in the dark for 75 min, before being placed on ice. All batches of nanostars being prepared were mixed together, and the absorbance spectrum of the nanostars was characterized to confirm the primary spectral peak which should be within the 760 nm to 780 nm region. Following initial absorbance characterization, the nanostars were subdivided into two cohorts: *para*-mercaptobenzoic acid (pMBA) (TCI America) and 5,5-dithiobis(2-nitrobenzoic acid) (DTNB) (TCI America). These labels were prepared in biomolecular-grade ethanol (Thermo Fisher) at a concentration of 2 mM and were added at a ratio of 15 µL of label solution per 10 mL of nanostar solution for pMBA and 20 µL of label solution per 10 mL of nanostar solution for DTNB. The thiol–gold conjugation was carried out at 4 °C for 15 min with gentle rotation. After this reaction, the absorbance of the labeled nanostars was measured to confirm an 8–12 nm redshift in the primary spectral peak. The labeled nanostars were then centrifuged at 3620 × *g* for 20 min and the supernatant was taken out. The supernatant was then removed and spun again to enhance recovery. The concentration of the nanostars was adjusted to approximately 0.175 mg mL^−1^ based on the absorbance measurements. The antibodies-linker conjugates that had reacted overnight were then added to the diluted nanostars at a ratio of 55 µL of antibody-linker solution for each 5.625 mL of diluted nanostar solution. The anti-VEGFR2 conjugate was added to the pMBA-labeled nanostars to create nanostar-pMBA-VEGFR2 probes, and anti-CD8a was added to the DTNB-labeled nanostars to generate nanostar-DTNB-CD8a probes. This solution was then reacted for 20–24 h on a rotating inverter at 4 °C.

Following this, the nanostar-antibody conjugates were diluted by 50% with milliQ water and separated into smaller aliquots of 10–12 mL. The solution was centrifuged at 1610 × *g* for 10 min to remove any unbound nanostars and excess reagents. The supernatant was then removed and spun again twice more to enhance recovery. The final conjugates were characterized by absorbance to confirm a 20–25 nm redshift. The quality of the nanoprobes was further evaluated by measuring the hydrodynamic diameter (estimated size) and zeta potential (surface charge) using a Zetasizer. SERS spectra were obtained by plating a droplet of the nanostar solution onto a 20 mm calcium fluoride (CaF2) substrate and acquiring measurements using a 5× Lexica objective with a 10-second acquisition per point and 5 replicate points per measurement.

### Cell viability assay

Cell viability was assessed using Cell Counting Kit-8 (CCK-8) assays obtained from Dojindo Laboratories. In each well of a 96-well plate, 5000 4T1 cells, quantified using a cell counter, were seeded with 100 µL of cRPMI, while one column was reserved for background subtraction. Following a 16 h incubation period, the culture medium was replaced. Control columns received a fresh untreated medium, whereas the remaining columns were treated with different concentrations of MGNs. After 24 h of exposure to the MGNs, 10 µL of the CCK-8 assay reagent was added to each well, and the mixture was incubated for 90 min in a dark environment. The plate was further incubated at room temperature for 25 min to stabilize the luminescent signal. Subsequently, the absorbance of each well at 450 nm was measured using a SpectaMax M3 microplate reader. All measurements were performed using three independent biological replicates (*n* = 3), with each biological replicate measured in technical triplicate. Technical replicates were averaged prior to statistical analysis, and results are reported as mean ± standard deviation.

### Preparation of cancer cells and tumor inoculation

4T1 murine cells were used for tumor inoculation. For 4T1 cells, the culture medium was prepared using RPMI 1640 (Gibco), supplemented with 10% sterile filtered FBS (Sigma-Aldrich) and 1% penicillin–streptomycin (Sigma-Aldrich) to produce complete RPMI medium (cRPMI). Cells were grown in T-75 flasks and incubated in a humidified incubator at 37 °C with an atmosphere of 5% CO_2_. To subculture, cells were washed with warmed sterile Dulbecco's phosphate-buffered saline (DPBS) and detached using 4 mL of warmed 0.25% trypsin-EDTA (Sigma). The trypsinization time was 4–5 min. After detachment, the trypsin-EDTA was neutralized by addition of 6–7 mL of warmed complete media. The cell suspension was then centrifuged at 250 × *g* for 4 min, and the supernatant was discarded. The cells were resuspended in 0.5 mL of warmed DPBS. Cell count was conducted using an Invitrogen Countess II automated cell counter using Trypan blue dye and a double-chamber cell counter slide (Invitrogen). The average of the two chambers was recorded as the cell count, and the cells were diluted with DPBS to an appropriate concentration. The cells were placed on ice for immediate inoculation. 4T1 cells were inoculated at a density of 0.5 million cells in 100 µL of DPBS. Inoculations were performed subcutaneously into the right flank of each mouse. Tumor growth was monitored daily, with measurements of tumor size and body weight recorded until the termination of the study.

### *In vivo* SERS imaging

Tumor site hair was removed using Nair hair removal cream, applied for 1 minute, and then washed off. *In vivo* SERS imaging was performed while the mice were under 2.5% isoflurane anesthesia and placed on a heated pad set to 37 °C. Imaging was performed in high confocal mode, with the focal point positioned 100 µm below the tumor surface. A 5× Leica objective with 0.12 numerical aperture was used, and 10 points were measured across the tumor. Each SERS scan involved five accumulations of 20 s (totaling 100 s) to ensure high SNR measurements. Per the IACUC protocol, the maximum anesthesia session length was 40 min, but all imaging sessions were completed within 30–32 min. For SERS nanoprobe imaging, mice received intraperitoneal injections of aqueous nanoprobes: 100 µL of 6 mg mL^−1^ nanoprobes (totaling 0.6 mg). Injections were spaced 30 min apart. SERS imaging was repeated at 6 h, 20 h, and 50 h post-injection. The collected SERS spectra were processed by applying a Savitzky–Golay filter, followed by background subtraction. The processed spectra were then normalized to the 1448 cm^−1^ band, corresponding to structural biomolecules such as proteins and lipids. Subsequently, data were further normalized to the background tissue signal for each Raman label (1076 cm^−1^ for aVEGFR2–pMBA and 1331 cm^−1^ for aCD8-DTNB).

### Dose dependent responder treatment study in murine tumors

For the treatment study, 0.5 million 4T1 cells were inoculated subcutaneously in each BALB/c mouse at the age of 6–7 weeks. Treatment began once tumors reached approximately 120 mm^3^ in size. The 7.5 µg dose treatment cohort received a combination of 7.5 µg ADU-S100 (Chemie Tek), the STING agonist, and 7.5 µg ODN2395 (Invivogen), the TLR9 agonist, administered in a total volume of 30 µL *via* intratumoral injections every other day for three doses on days 13, 15, and 17. The 5 µg dose treatment cohort received a combination of 5 µg ADU-S100 and 5 µg ODN2395. In contrast, the PBS control cohort was administered 30 µL of PBS in a staggered schedule on days 13, 15, and 17 due to anticipated tumor growth exceeding humane limits by day 20. Two days after the final treatment dose, mice were either euthanized, and tumor tissue was collected for *ex vivo* analysis or injected with nanostars for *in vivo* imaging. For the nonresponder treatment study, the same regimen was followed, with the treated cohort receiving 7.5 µg antiOX40 (Bio X cell, clone: OX-86) *via* intratumoral injections.

### Flow cytometry sample preparation

Following euthanasia, tumor tissues were harvested and processed for single-cell flow cytometric analysis. Excised tumors were cut into pieces using sterile scalpels and enzymatically digested in 1 mL of pre-warmed DMEM supplemented with 4.4 mg mL^−1^ collagenase. Digestion was carried out at 37 °C for 30 min on a rotator. The reaction was quenched with 0.7 mL of ice-cold 10 mM EDTA in DPBS, followed by mechanical dissociation using a bead homogenizer for two 60-second cycles. The resulting cell suspension was filtered through a cell strainer and washed with 4 mL of ice-cold EDTA-DPBS. Cells were pelleted by centrifugation at 500 × *g* for 5 min at 4 °C and resuspended in 1 mL of ice-cold EDTA-DPBS. Viable cell counts were determined using a Countess II Automated Cell Counter (Invitrogen) with Trypan Blue exclusion. For each assay, one million viable cells were allocated. Two distinct antibody panels were used per tumor: one targeting T cell and VEGF-associated markers, and another for regulatory T cell (*T*_reg_) profiling.

The T cell & VEGF cell panel included FITC anti-rat IgG as the secondary antibody for VEGF, PE anti-mouse CD209a (Biolegend, clone: MMD3), PE/Cyanine7 anti-mouse CD4 (Biolegend, clone: GK1.5), APC anti-mouse CD274 (Biolegend, clone: 10F.9G2), APC/Cyanine7 anti-mouse CD3 (Biolegend, clone: 17A2), Brilliant Violet 711 anti-mouse CD8b (Biolegend, clone: YTS156.7.7), Brilliant Violet 785 anti-mouse CD11c (Biolegend, clone: N418) and Zombie Aqua (Biolegend) dye for viability assessment. The *T*_reg_ panel consisted of FITC anti-mouse CD25 (Biolegend, clone: PC61), PE/Cyanine7 anti-mouse CD4, APC anti-mouse FoxP3 (eBioscience, clone: FJK-16s), APC/Cyanine7 anti-mouse CD3 and Zombie Aqua dye.

Prior to surface staining, Fc receptors were blocked with 1 µg of anti-mouse CD16/32 (BioLegend, clone: 93) to prevent nonspecific antibody binding. Samples were first incubated with Zombie Aqua dye at room temperature for 5 min. Surface antibodies were then added, and cells were incubated at 4 °C for 30 min. Following staining, cells were washed and centrifuged at 500 × *g* for 5 min at 4 °C. For samples stained with the T cell/VEGF panel, cells were fixed in methanol-free 4% paraformaldehyde for 5 min at 4 °C, followed by centrifugation and resuspension in ∼150 µL of FACS buffer. For intracellular staining of FOXP3 in the *T*_reg_ panel, surface-stained cells were incubated with 1 mL of FOXP3 fixation/permeabilization buffer (eBioscience) at room temperature for 30 min. Cells were then washed with 1× permeabilization buffer, centrifuged, and resuspended in ∼100 µL of the same buffer. Subsequently, 1 µg of anti-FOXP3 antibody was added, and the samples were incubated for 30 min at room temperature. After an additional wash and centrifugation step, cells were resuspended in ∼150 µL FACS buffer for acquisition. All flow cytometry measurements were performed at the Iowa State University Flow Cytometry Facility, and data were analyzed using FlowJo software.

### *Ex vivo* immunofluorescence imaging

Following necropsy, a portion of each tumor was embedded in optimal cutting temperature (OCT) compound and snap-frozen. Tissue sections of 8 µm thickness were prepared using a cryostat and mounted onto poly-l-lysine–coated glass slides (electron microscopy sciences). Sections were fixed in ice-cold methanol for 10 seconds and subsequently washed twice with pre-warmed PBS (37 °C) for 5 min to remove residual OCT. A blocking step was performed at room temperature for 30 min using PBS supplemented with 0.1% Tween-20, 1% BSA (Sigma), and 22.52 mg mL^−1^ glycine (Sigma). After blocking, slides were incubated overnight at 4 °C with primary antibodies conjugated to fluorescent dyes, diluted in PBST (0.1% Tween-20 in PBS). The antibody panel included FITC-conjugated anti-mouse CD8a (BioLegend, clone: 53-6.7), Alexa Fluor 647–labeled STING/TMEM173 (R&D Systems), Alexa Fluor 647–labeled anti-mouse Ki67 (BioLegend, clone: 16A8) and FITC-labeled CD31 (BioLegend; clone: 390). The following day, sections were washed five times in PBST for 5 min each. Nuclear staining was performed using ProLong™ Gold Antifade Mountant with DAPI (Invitrogen), and coverslips were applied. Slides were stored at 4 °C overnight to allow complete curing. Fluorescent imaging was performed using an Echo Revolve microscope at 20× magnification. Quantitative analysis of fluorescence signal intensity was conducted using ImageJ.

### Enzyme-Linked Immunosorbent Assay (ELISA)

After isolating serum from the blood collected from treated mice post-euthanasia on the eighteenth day, ELISA analysis for type 1 interferons and TNF-α was performed using the following uncoated ELISA kits according to the manufacturer’s protocols mouse IFN-alpha/beta R2 ELISA Kit (Thermo Fisher Scientific, cat: EM39RB), mouse TNF alpha (Thermo Fisher Scientific, cat: 88-7324-88), mouse IL-6 (Thermo Fisher Scientific, cat: 88-7064-88) and mouse IFNg (Thermo Fisher Scientific, cat: 88-8314-88). The ELISA concentrations were all normalized to their corresponding total protein concentrations using a BCA protein assay kit.

### Statistical analysis

Statistical significance was determined using a two-tailed heteroscedastic Student's *t*-test and defined as *: *p* < 0.05, **: *p* < 0.01, ***: *p* < 0.001, and n.s. indicates not significant.

## Author contributions

R.B. conceived the project idea, directed the study design, provided guidance throughout the study, and edited the manuscript. SK performed all nanomaterial synthesis, cell experiments, animal care and experiments, data analysis, data interpretation, and wrote the manuscript. GC and AM assisted in *in vivo* imaging experiments, and sample preparation for *ex vivo* experiments. GC trained SK in nanomaterial synthesis, Raman analysis and Raman measurement. CZ assisted in nanomaterial synthesis.

## Ethical statement

All animal procedures were performed in accordance with the Guidelines for Care and Use of Laboratory Animals of Iowa State University, Ames, IA, USA and approved by the Animal Ethics Committee of Institutional Animal Care and Use Committee (IACUC) under protocol number IACUC-23-098.

## Conflicts of interest

The authors declare no conflict of interest.

## Supplementary Material

NH-011-D5NH00687B-s001

## Data Availability

The data supporting this study's findings will be made available upon request from the corresponding author. Supporting information (SI): characterization of MGNs functionalized with mPEG; CCK-8 cell viability assay in 4T1 cells after STING + TLR9 treatment; CT imaging and ROI of STING + TLR9 treated cohort; tumor weight, spleen weight, immunofluorescence of CD31, and flow cytometry of CD4^+^ T cells for STING + TLR9 treatment; FACS gating schema; tumor weight, spleen weight, immunofluorescence of CD31, and flow cytometry of CD4^+^ T cells for antiOX40 treatment; correlation coefficient values for the heatmap comparing marker pairs from *ex vivo* Raman and IF imaging. See DOI: https://doi.org/10.1039/d5nh00687b.
