# Supplementary material for: Simultaneous detection of lymphocytes and tumor cells in vivo in response to STING-TLR9 immunotherapy with Raman active multiplexed gold nanostars
Source: Nanoscale Horiz. 2026 Jan 7;11(3):865–82. doi: 10.1039/d5nh00687b (PMC12810492; doi:10.1039/d5nh00687b)
Supplement: NH-011-D5NH00687B-s001 [file NH-011-D5NH00687B-s001.pdf]

Supplementary Information (SI) for Nanoscale Horizons.
This journal is © The Royal Society of Chemistry 2026

Simultaneous detection of lymphocytes and tumor cells *in vivo* in response to STING-TLR9 immunotherapy with Raman active multiplexed gold nanostars

Siddhant Kothadiya[a,b], Gabe Cutshaw[a,b], Ansuja Mathew[a,b], Casey Zielinski[a] and Rizia Bardhan[a,b,*]

[a]Department of Chemical and Biological Engineering, Iowa State University, Ames, IA 50011, USA
[b]Nanovaccine Institute, Iowa State University, Ames, IA 50012, USA

*Corresponding author: rbardhan@iastate.edu

Table of Contents

Figure S1. Characterization of multiplexed gold nanostars (MGNs) functionalized with mPEG……………………………………………………………………………………..2

Figure S2. Shelf-life stability of MGNs during storage………………………………………...2

Figure S3. Concentration dependent CCK-8 cell viability assay in 4T1 cells after 24 hours of treatment with STING+TLR9……………………………………………………………………..3

Figure S4. Computed tomography (CT) imaging and ROI of STING+TLR9 treated cohort…….3

Figure S5. Tumor weight, spleen weight, representative 20× IF images for CD31 and flow cytometry analysis of $CD4^+$ T cells for the STING+TLR9 combination immunotherapy cohort.…4

Figure S6. FACs gating strategy shown for control tumors, for CD274+ VEGFR2+ cells, CD8+ T-cells and CD4+ T-cells, CD11c+ CD209- dendritic cells………………………………………5

Figure S7. FACs gating strategy shown for control tumors, for FOXP3+ CD25+ ………………..5

Figure S8. Corresponding raw, unprocessed IF images for Figure 4 f, g in main text……………..6

Figure S9. Tumor weight, spleen weight representative 20× IF images for CD31 and flow cytometry analysis of $CD4^+$ T cells for the antiOX40 monotherapy cohort.………………………7

Figure S10. Corresponding raw, unprocessed IF images for Figure 4 f, g in main text…………..8

Table S1. Shelf-life characterization of MGNs during storage FWHM and DLS values…………9

Table S2. Correlation coefficient values for heatmap comparing marker pairs quantified by *ex vivo* Raman and IF imaging………………………………………………………………………9

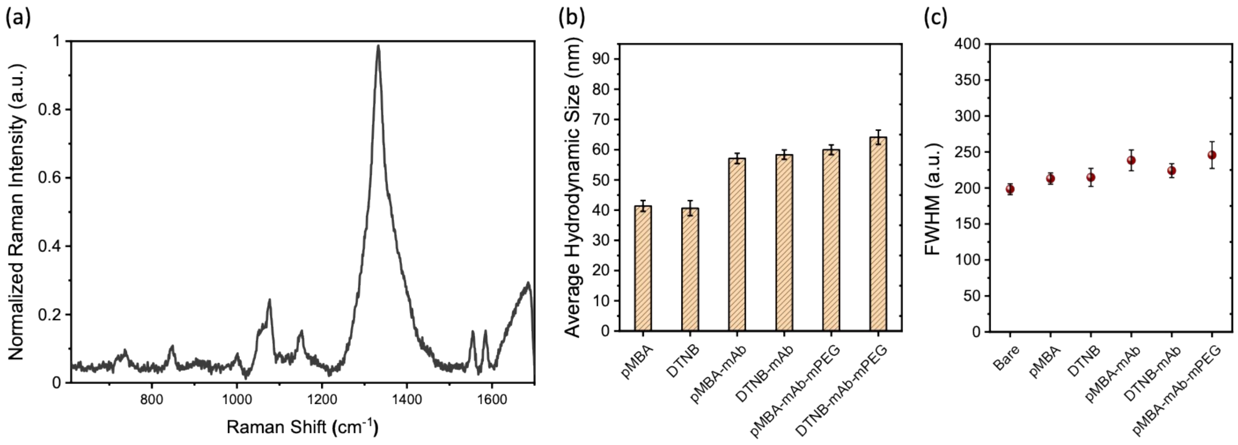

Figure S1. Characterization of multiplexed gold nanostars (MGNs) functionalized with mPEG. (a) SERS spectra of MGNs (GNs/mAb/pMBA + GNs/mAb/DTNB at a 1:1 ratio) targeting both DTNB (1331 cm^{-1}) and pMBA (1076 cm^{-1}) reporters respectively. (b) Hydrodynamic size of MGNs before and after functionalization with mPEG and the (c) corresponding full width at half maximum (FWHM) of the extinction of the same samples.

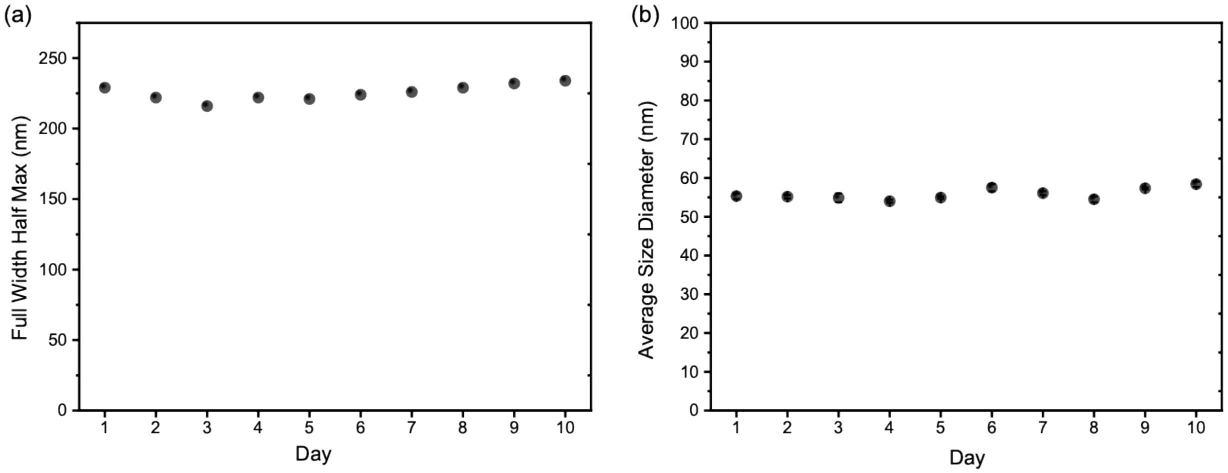

Figure S2. Shelf-life stability of MGNs during storage. MGNs were aliquoted and stored in water at 4 °C, and stability was monitored over a 10-day period. (a) Full width at half maximum (FWHM) of the localized surface plasmon resonance extinction peak measured with UV–Vis-NIR and (b) hydrodynamic diameter at each time point measured with DLS, showing minimal variation over time.

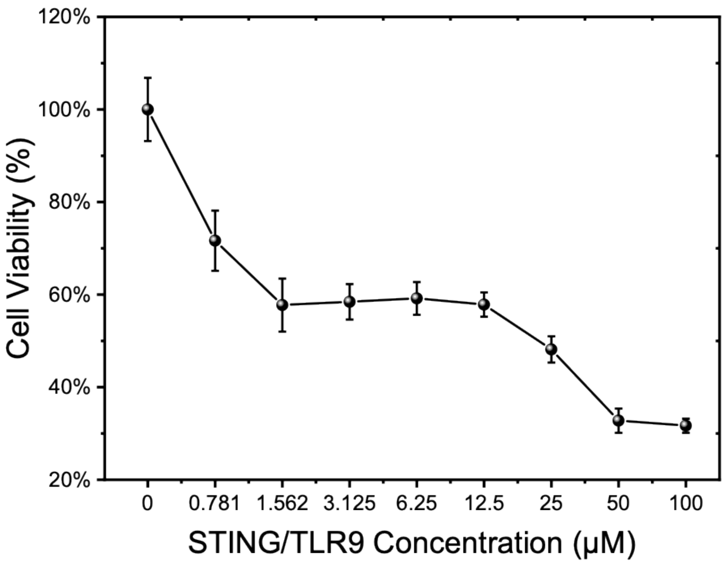

Figure S3. Concentration dependent CCK-8 cell viability assay in 4T1 cells after 24 h of treatment with STING+TLR9 combination immunotherapy.

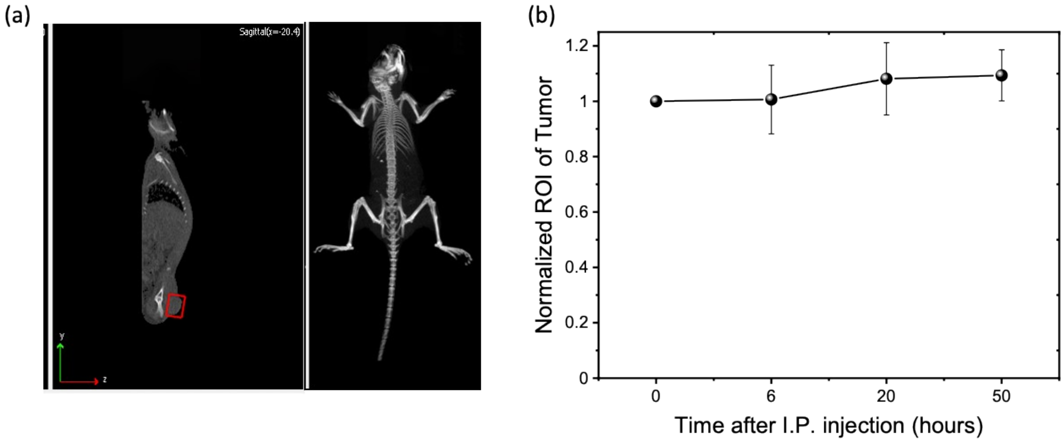

Figure S4. (a) Representative sagittal and longitudinal imaging of mice 6 h after MGNs injection (b) Longitudinal analysis of normalized ROI of the tumor over the duration of the in vivo experiments.

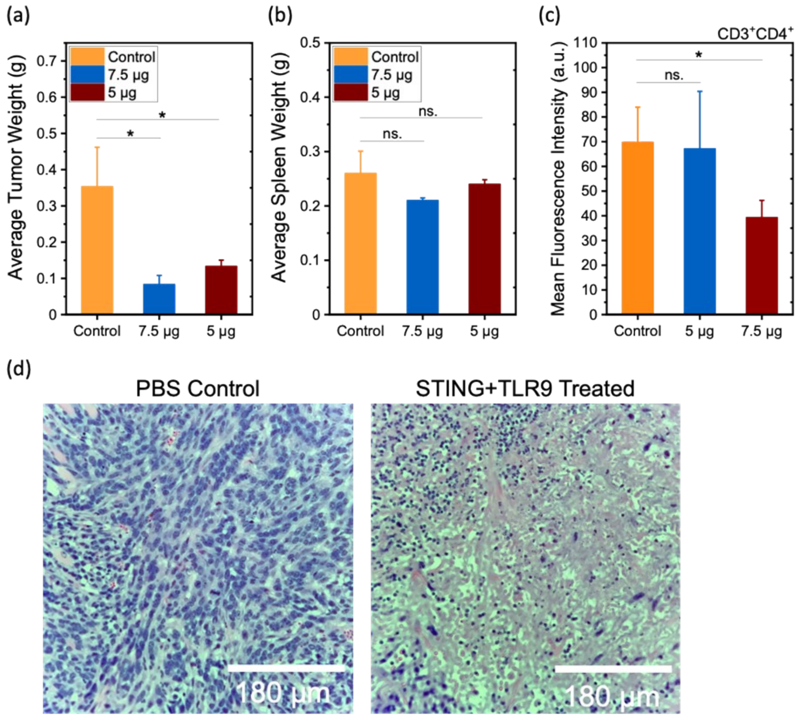

Figure S5. Treatment outcomes for mice under STING+TLR9 combination immunotherapy. (a) Mean mouse tumor weights at end of study for PBS control (yellow, n = 3), 7.5 µg STING+TLR9 immunotherapy (blue, n = 3), and 5 µg STING+TLR9 immunotherapy (red, n = 3) cohorts. (b) Mean mouse spleen weights at end of study for PBS control (yellow, n = 3), 7.5 µg STING+TLR9 immunotherapy (blue, n = 3), and 5 µg STING+TLR9 immunotherapy (red, n = 3) cohorts. (c) Flow cytometry analysis of $CD3^{+}CD4^{+}$ T cells for PBS control (yellow, n = 3), 7.5 µg STING+TLR9 immunotherapy (blue, n = 3), and 5 µg STING+TLR9 immunotherapy (red, n = 3) cohorts. (d) Representative H&E images for PBS control and STING+TLR9 treated cohorts.

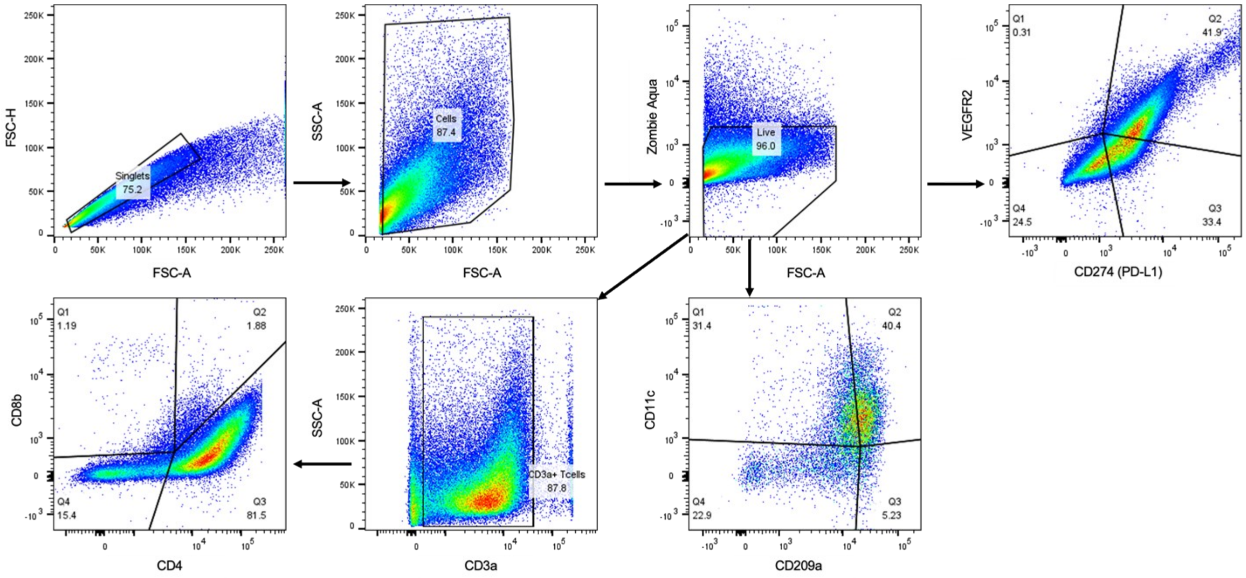

Figure S6. FACs gating strategy shown for control tumors, for CD274^{+} VEGFR2^{+} cells, CD8^{+} T-cells and CD4^{+} T-cells, CD11c^{+} CD209^{-} dendritic cells.

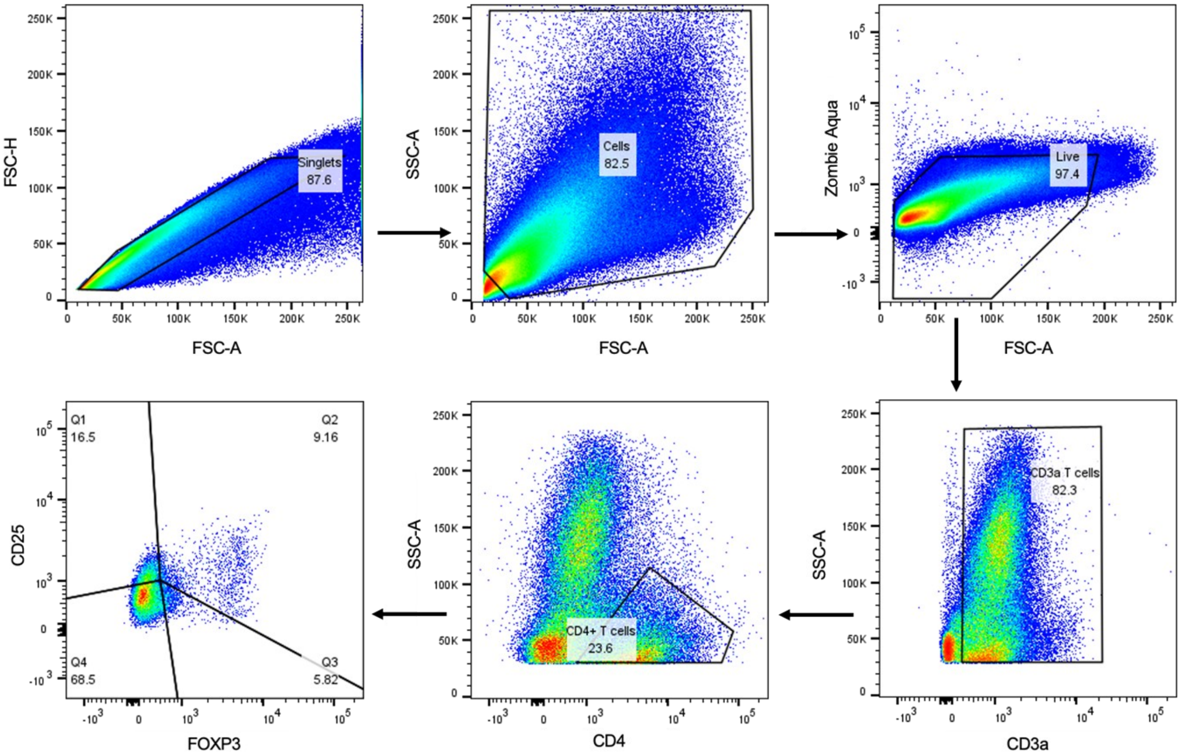

Figure S7. FACs gating strategy shown for control tumors, for FOXP3^{+} CD25^{+}

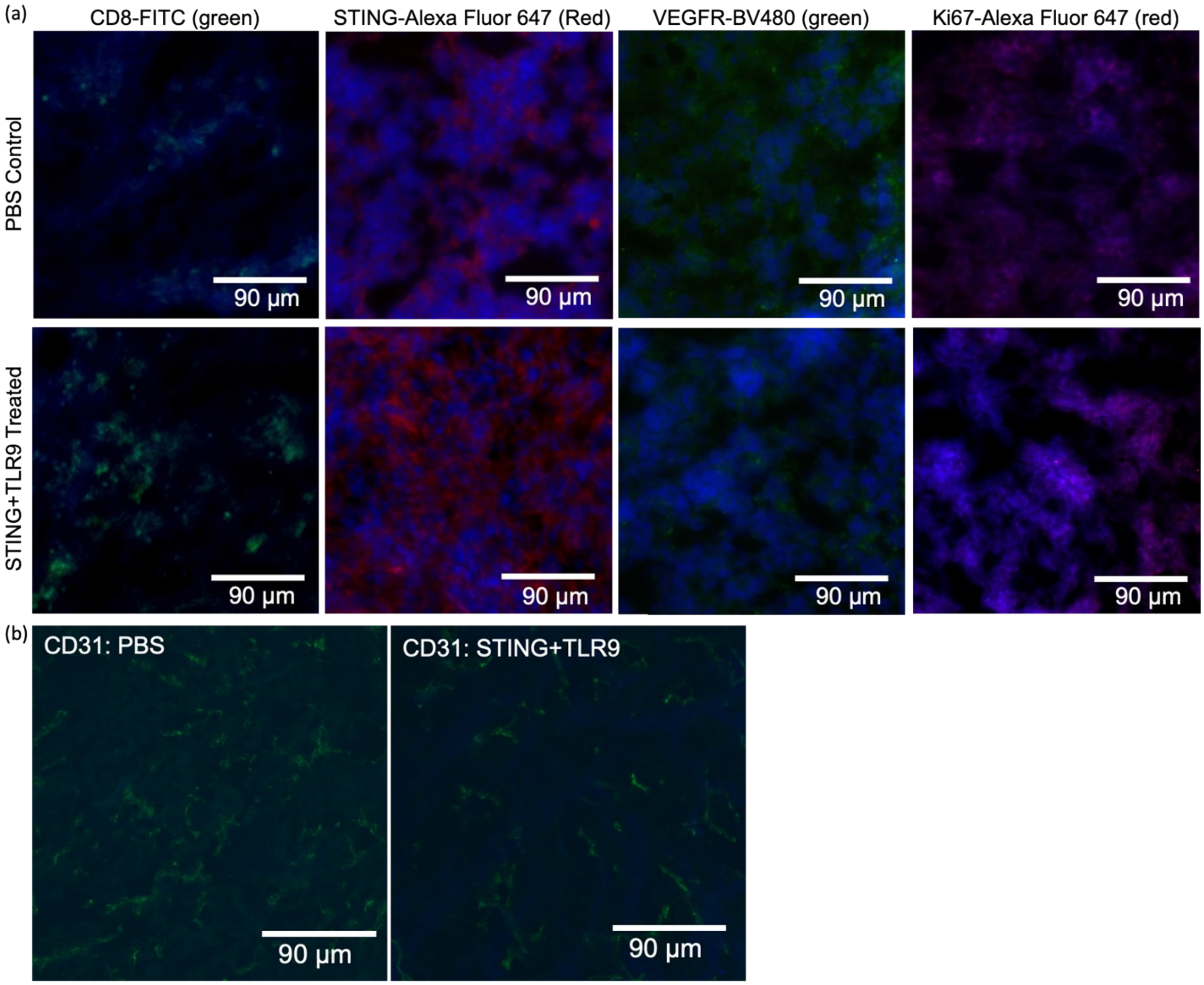

Figure S8. Corresponding raw, unprocessed IF images for Figure 4 f, g in the main text.

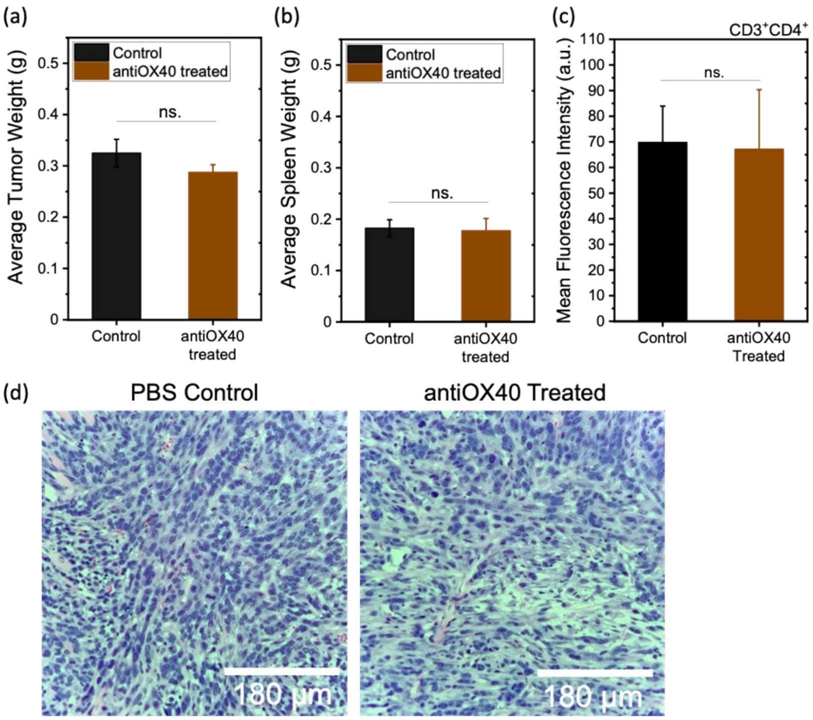

Figure S9. Treatment outcomes for mice under antiOX40 monotherapy. (a) Mean mouse tumor weights at end of study for PBS control (black, n = 3), and antiOX40 monotherapy (brown, n = 3) cohorts. (b) Mean mouse tumor weights at end of study for PBS control (black, n = 3), and antiOX40 monotherapy (brown, n = 3) cohorts. (c) Flow cytometry analysis of $CD3^{+}CD4^{+}$ T cells or PBS control (black, n = 3), and antiOX40 monotherapy (brown, n = 3) cohorts. (d)Representative H&E images for PBS control and antiOX40 treated cohorts.

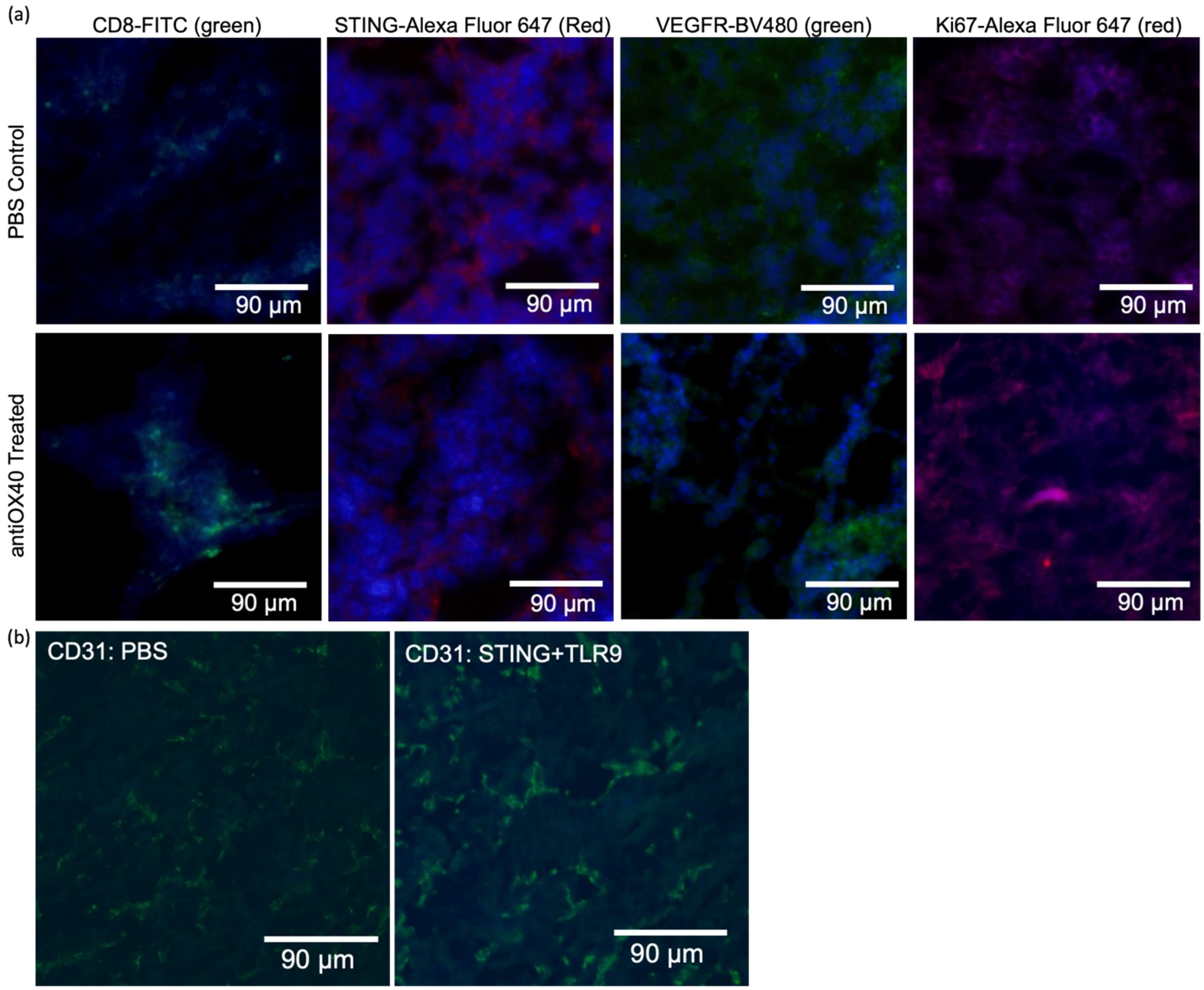

Figure S10. Corresponding raw, unprocessed IF images for Figure 5l, m in the main text.

Table S1. Shelf-life characterization of MGNs during storage. Full width at half maximum (FWHM) of the localized surface plasmon resonance peak obtained from UV–Vis extinction measurements and hydrodynamic diameter measured by DLS for MGNs stored in water at 4°C over a 10-day period.

Day	FWHM (nm)	Average Size Diameter (nm)
1	229	55.31 ± 0.71
2	222	55.17 ± 0.45
3	216	54.87 ± 1.14
4	222	54 ± 0.38
5	221	54.93 ± 0.31
6	224	57.5 ± 0.91
7	226	56.1 ± 0.49
8	229	54.5 ± 0.75
9	232	57.33 ± 0.25
10	234	58.4 ± 0.67

Table S2. Correlation coefficient values for heatmap comparing marker pairs quantified by *ex vivo* Raman and IF imaging for (a) STING+TLR9 (n = 3) and (b) antiOX40 (n = 3) treated cohorts, associated with Figure 6i; Inverse correlation is plotted in blue while joint correlation is plotted in red.

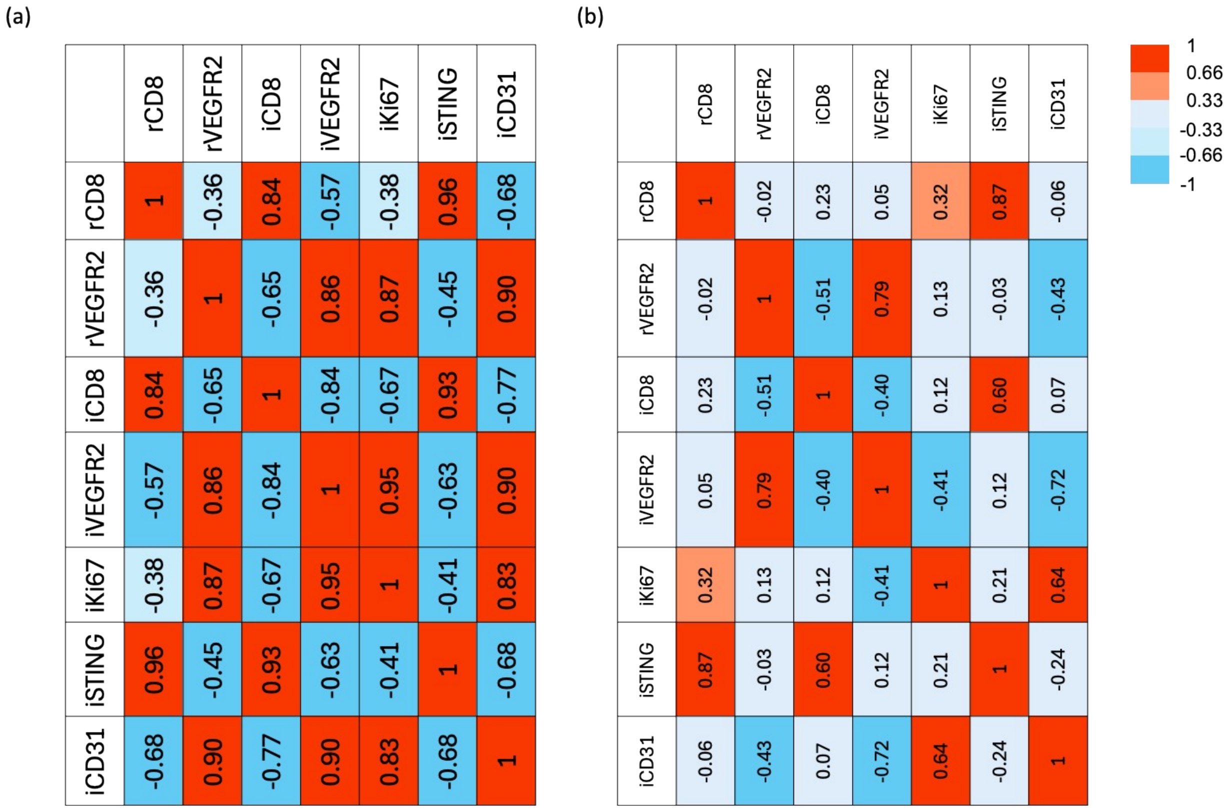

(a)

	rCD8	rVEGFR2	iCD8	iVEGFR2	iKi67	iSTING	iCD31
rCD8	1	-0.36	0.84	-0.57	-0.38	0.96	-0.68
rVEGFR2	-0.36	1	-0.65	0.86	0.87	-0.45	0.90
iCD8	0.84	-0.65	1	-0.84	-0.67	0.93	-0.77
iVEGFR2	-0.57	0.86	-0.84	1	0.95	-0.63	0.90
iKi67	-0.38	0.87	-0.67	0.95	1	-0.41	0.83
iSTING	0.96	-0.45	0.93	-0.63	-0.41	1	-0.68
iCD31	-0.68	0.90	-0.77	0.90	0.83	-0.68	1

(b)

	rCD8	rVEGFR2	iCD8	iVEGFR2	iKi67	iSTING	iCD31
rCD8	1	-0.02	0.23	0.05	0.32	0.87	-0.06
rVEGFR2	-0.02	1	-0.51	0.79	0.13	-0.03	-0.43
iCD8	0.23	-0.51	1	-0.40	0.12	0.60	0.07
iVEGFR2	0.05	0.79	-0.40	1	-0.41	0.12	-0.72
iKi67	0.32	0.13	0.12	-0.41	1	0.21	0.64
iSTING	0.87	-0.03	0.60	0.12	0.21	1	-0.24
iCD31	-0.06	-0.43	0.07	-0.72	0.64	-0.24	1
